# Differentiation of Zebrafish Melanophores Depends on Transcription Factors AP2 Alpha and AP2 Epsilon

**DOI:** 10.1371/journal.pgen.1001122

**Published:** 2010-09-16

**Authors:** Eric Van Otterloo, Wei Li, Gregory Bonde, Kristopher M. Day, Mei-Yu Hsu, Robert A. Cornell

**Affiliations:** 1 Department of Anatomy and Cell Biology, University of Iowa, Iowa City, Iowa, United States of America; 2 Interdisciplinary Graduate Program in Genetics, University of Iowa, Iowa City, Iowa, United States of America; 3 Department of Pathology, Program in Dermatopathology, Brigham and Women's Hospital, Boston, Massachusetts, United States of America; Stanford University, United States of America

## Abstract

A model of the gene-regulatory-network (GRN), governing growth, survival, and differentiation of melanocytes, has emerged from studies of mouse coat color mutants and melanoma cell lines. In this model, Transcription Factor Activator Protein 2 alpha (TFAP2A) contributes to melanocyte development by activating expression of the gene encoding the receptor tyrosine kinase Kit. Next, ligand-bound Kit stimulates a pathway activating transcription factor Microphthalmia (Mitf), which promotes differentiation and survival of melanocytes by activating expression of Tyrosinase family members, *Bcl2*, and other genes. The model predicts that in both *Tfap2a* and *Kit* null mutants there will be a phenotype of reduced melanocytes and that, because Tfap2a acts upstream of Kit, this phenotype will be more severe, or at least as severe as, in *Tfap2a* null mutants in comparison to *Kit* null mutants. Unexpectedly, this is not the case in zebrafish or mouse. Because many Tfap2 family members have identical DNA–binding specificity, we reasoned that another Tfap2 family member may work redundantly with Tfap2a in promoting *Kit* expression. We report that *tfap2e* is expressed in melanoblasts and melanophores in zebrafish embryos and that its orthologue, *TFAP2E,* is expressed in human melanocytes. We provide evidence that Tfap2e functions redundantly with Tfap2a to maintain *kita* expression in zebrafish embryonic melanophores. Further, we show that, in contrast to in *kita* mutants where embryonic melanophores appear to differentiate normally, in *tfap2a/e* doubly-deficient embryonic melanophores are small and under-melanized, although they retain expression of *mitfa*. Interestingly, forcing expression of *mitfa* in *tfap2a/e* doubly-deficient embryos partially restores melanophore differentiation. These findings reveal that Tfap2 activity, mediated redundantly by Tfap2a and Tfap2e, promotes melanophore differentiation in parallel with Mitf by an effector other than Kit. This work illustrates how analysis of single-gene mutants may fail to identify steps in a GRN that are affected by the redundant activity of related proteins.

## Author Summary

Neural crest-derived pigment cells, known as melanocytes, are important to an organism's survival because they protect skin cells from ultraviolet radiation, camouflage the organism from predators, and contribute to sexual selection. Networks of regulatory proteins control the steps of melanocyte development, including lineage specification, migration, survival, and differentiation. Gaps in our understanding of these networks hamper progress in effective prevention and treatment of diseases of melanocytes, including metastatic melanoma and vitiligo. Studies conducted in tissue-culture cells and mouse embryos implicate regulatory proteins including the transcription factor TFAP2A, the growth factor receptor KIT, and the transcription factor MITF as being important for multiple steps in melanocyte development. Abnormalities in *TFAP2A*, *KIT,* and *MITF* expression in melanoma highlight the importance of this pathway in human disease. Here we show that a gene closely related to *TFAP2A*, *tfap2e*, is expressed in zebrafish embryonic melanocytes and human melanocytes. We provide evidence that Tfap2e cooperates with Tfap2a to promote expression of zebrafish *kita* in embryonic melanocytes. Further we show that an effector of Tfap2a/e activity other than Kita is required for melanocyte differentiation and that this effector acts upstream or in parallel with Mitfa activity. These findings reveal unexpected complexity to the gene-regulatory network governing melanocyte differentiation.

## Introduction

An important participant in the gene-regulatory-network (GRN) that governs the differentiation of melanocytes from neural crest precursors (i.e., the melanocyte GRN) is the class III receptor tyrosine kinase Kit. In mouse embryos, binding of this growth-factor receptor by its ligand, stem cell factor (SCF), promotes the growth, survival, migration, and possibly terminal differentiation of melanocytes [1]. Mouse embryos homozygous for hypomorphic alleles of *Kit* completely lack melanocytes (embryos homozygous for *Kit* null alleles die prior to pigmentation) [2]–[6]. While ligand-bound Kit stimulates many signal transduction pathways, its effects on melanocyte growth and differentiation appear to occur via the Ras/Raf/Map Kinase pathway. Activity of this pathway results in phosphorylation of Microphthalmia transcription factor (Mitf); phosphorylation of Mitf regulates its activity and stability [7], [8]. Within melanoblasts, Mitf promotes a) cell-cycle exit, by activating expression of the *p21^WAF1^*, a cyclin-dependent kinase inhibitor [9], b) cell survival, by upregulating the expression of *BCL2* [10], and c) melanin synthesis, by activating expression of *Tyrosinase* (*Tyr*), *Tyrosinase-related protein 1* (*Tyrp1*), and *Tyrosinase-related protein 2* (*Tyrp2*, also known as *Dopachrome tautomerase*, *Dct*) [11]–[14]. Thus, Kit signaling is essential for normal melanocyte development, at least in part via its ability to stimulate Mitf activity. Of note, *KIT* levels are reported to be lower in metastatic melanoma cell lines than in benign nevi, and forced expression of *KIT* in these cells has been shown to induce apoptosis [15]. These findings highlight the importance of understanding the regulation of *Kit* expression within the melanocyte lineage.

While there is evidence that the *KIT* gene is dependent on direct stimulation by the Transcription Factor Activator Protein 2 alpha (TFAP2A) in melanoma, analyses of mutant model organisms indicate a more complex regulatory scenario within embryonic melanocytes. TFAP2A and other members of the TFAP2 family control cell fate specification, cell differentiation, cell survival and cell proliferation within neural crest, skin, breast epithelium, and other embryonic cell types and stem cells [16], [17]. Gel shift experiments showed that TFAP2A can bind an element 1.2 kb upstream of the *KIT* transcription start site, and expression driven by this enhancer in melanoma cells is lost when the TFAP2 binding sites are deleted [18]. Moreover, forced expression of the TFAP2A DNA binding domain, which presumably unseats endogenous TFAP2A and thus acts as a dominant negative AP2, prevents expression of *KIT* in these cells [18]. Mice lacking the *Tfap2a* gene do not live long enough to develop melanocytes, due to failure of body wall closure [19], [20]. However, in embryos with Wnt1-CRE-mediated deletion of *Tfap2a* specifically within the neural crest, melanocytes are absent from the belly [21]. Interestingly, this phenotype resembles that of heterozygous, not homozygous, *Kit* loss-of-function mutants, suggesting that loss of *Tfap2a* leads to a reduction rather than complete loss of *Kit* expression. Zebrafish have two orthologues of mammalian *Kit*, known as *kita* and *kitb*; only *kita* is expressed in the melanophore lineage [22]. In *kita* homozygous null mutants (i.e., *kita* mutants) relative to their wild-type counterparts, embryonic melanophores are reduced in number by about 40%, migrate less, and eventually undergo apoptosis [23]. In zebrafish *tfap2a* homozygous null mutants (i.e., *tfap2a* mutants), *kita* expression is reduced and embryonic melanophores exhibit reduced migration [24], [25]. However, in contrast to the melanophores in *kita* mutants, those in *tfap2a* mutants do not appear to die, at least as long these animals survive [23], [26]. The simplest explanation for this difference is that *kita* expression in melanophores is initially dependent on *tfap2a* but later becomes independent of it. How can the dominant negative AP2 block *Kit* expression while loss of Tfap2a only diminishes or delays it? Because many Tfap2 family members have the same DNA binding affinity, it is possible that another such family member cooperates with Tfap2a to activate *Kit* expression.

Here we show that Tfap2e, a homolog of Tfap2a with the equivalent DNA binding specificity, is expressed in zebrafish melanoblasts and in cultures of primary human melanocytes. With single and double knockdown studies, we show that while Tfap2e is not required for the development of embryonic melanophores, it functions redundantly with Tfap2a in maintaining *kita* expression in embryonic melanophores. Interestingly, in contrast to the situation in *kita* mutants, the melanophores in embryos doubly deficient for *tfap2a/e* fail to differentiate. These results imply that Tfap2 activity has targets other than *kita* that are important for melanophore development. We find that forced expression of *mitfa* partially restores melanophores in embryos lacking *tfap2a* and *tfap2e*, implying that the targets of Tfap2a/e function to stimulate Mitfa activity or act in parallel with it. These findings reveal unexpected roles for Tfap2 activity in the melanocyte GRN.

## Results

### *tfap2e* is expressed in zebrafish melanoblasts and cultured human melanocytes

To determine if a second Tfap2 family member is expressed in the melanoblast lineage, we identified orthologues of *Tfap2b*, *Tfap2c, Tfap2d,* and *Tfap2e* in a database of expressed sequence tags (www.ensembl.org), amplified partial clones of at least 1 kb from each to make a probe for *in situ* hybridization, and examined the expression of each in embryos that ranged in stage from 0.5 hours post fertilization (hpf), revealing maternal expression, to 48 hpf. Expression patterns of *tfap2b* and *tfap2c* have previously been reported [27], [28]. We did not detect expression of *tfap2b*, *tfap2c,* or *tfap2d* in melanoblasts or melanophores (Figure S1), so we did not pursue these orthologues in the context of melanophore development.

**Figure 1:**
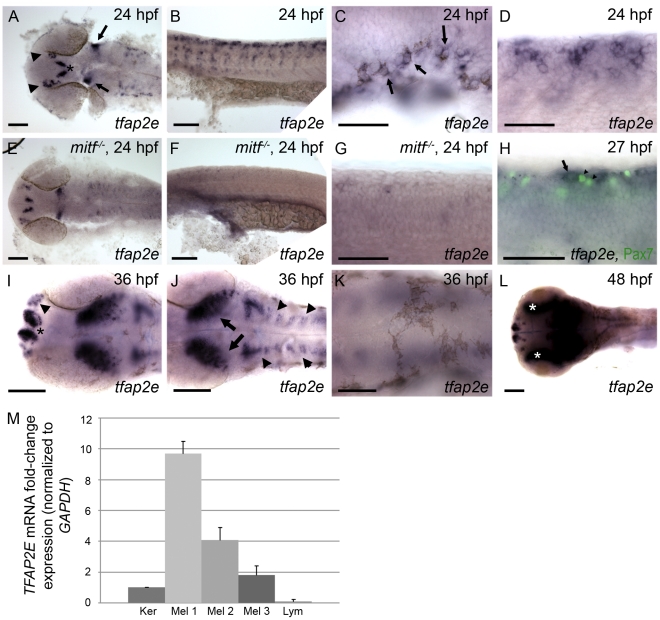
Characterization of *tfap2e* expression during embryogenesis. Wild-type zebrafish embryos, unless otherwise indicated, fixed at the stage indicated and processed to reveal *tfap2e* expression by RNA *in situ* hybridization. All embryos in this and subsequent figures are oriented with anterior to the left. (A) Dorsal view of the head showing *tfap2e* expression in presumed olfactory placode (arrowheads), medial telencephelon (asterisk), and hindbrain (arrows). (B) Lateral view of the trunk, showing *tfap2e* expression in cells migrating from the dorsal neural tube. (C) Lateral view just caudal to the ear. *tfap2e* expression is seen in newly-pigmented melanophores (arrows). (D) Higher-magnification view of the *tfap2e*-expressing cells of the trunk that are shown in panel B. (E–G) *tfap2e* expression in *mitfa^b692^* homozygous mutant embryos, in (E) dorsal and (F,G) lateral views. E) Expression of *tfap2e* in the head is virtually normal, (F, G) while its expression in the trunk is virtually absent. (H) Lateral view of a wild-type embryo processed to reveal *tfap2e* mRNA and Pax7 protein, a marker of xanthophores. *tfap2e* expression (arrow) does not overlap with α-Pax7 immunoreactivity (arrowheads). (I,J) Dorsal head views. I) At 36 hpf, *tfap2e* expression is visible in the olfactory placode (arrowheads in I), in bilateral clusters in the telencephalon (asterisk); J) in the optic tectum (arrows), and in rhombomeres (arrowheads in J). K, L) Dorsal views of the head. K) At 36 hpf expression of *tfap2e* in melanophores is no longer detectable. L) At 48 hpf *tfap2e* expression is detected in the retina (asterisks). (M) Quantitative RT-PCR shows that expression of *TFAP2E* in 3 independent primary human melanocytes (Mel 1–3) is about 2–10 fold higher than in keratinocytes (Ker), while its expression in Jurkat cells (lymphocytes, Lym) is about 10 fold lower than in keratinocytes. Scale bars: (A, B, E, F, H, I, K), 100 µm; (C, D, G, J), 50 µm.

In 8-cell zebrafish embryos, maternal *tfap2e* transcripts were detected by both *in situ* hybridization and semi-quantitative RT-PCR (not shown). At 24 hpf, *tfap2e* expression was detected in several regions of the brain, including presumed olfactory bulb, as in mouse embryos [29], [30] (Figure 1A), and also within dispersed cells in the trunk that we assumed to be a subset of migrating neural crest cells (Figure 1B and 1D). At this stage, *tfap2e* expression was detectable in early-differentiating melanophores close to the ear (Figure 1C), suggesting that the dispersed, non-melanized cells expressing *tfap2e* were melanoblasts. To test this possibility, we probed homozygous *mitfa* null mutant embryos (i.e., *mitfa^b692^*), which are devoid of melanoblasts [31], and found that *tfap2e* expression was absent from the dispersed cells in the trunk (Figure 1E-1G). This result was consistent with expression of *tfap2e* in melanoblasts. However, because *mitfa* is co-expressed with *xdh* and *fms*, two markers of xanthophore precursors [32], it was conceivable that *tfap2e* was expressed in the xanthophore lineage, in an Mitfa-dependent fashion. To test whether *tfap2e* is expressed in xanthophores, we processed embryos to simultaneously reveal expression of *tfap2e* mRNA and Pax7 protein, a marker of the xanthophore lineage [33]. We did not detect overlap of the two signals, which implies that *tfap2e* is not expressed in xanthophores (Figure 1H). In wild-type embryos at 36 hpf, *tfap2e* expression was present in the forebrain and presumed optic tectum, and expanded in the hindbrain relative to earlier stages (Figure 1I and 1J). However, at this stage expression was not detected in melanophores (Figure 1K). At 48 hpf, high-level *tfap2e* expression was also observed in the retina (Figure 1L).

To assess if melanocyte-specific expression of *TFAP2E* is conserved in humans, we performed quantitative RT-PCR on cDNA generated from various human cell lines. We detected higher levels of *TFAP2E* message in three independent isolates of primary melanocytes, consistent with microarray data indicating expression of *TFAP2E* in melanocytes and melanoma cell lines [34]. Expression in melanocytes was 2–10 fold higher than in a keratinocyte cell line, and approximately 50–100 fold higher than in a lymphocyte cell line (Figure 1M). In summary, *tfap2e* is expressed in zebrafish melanoblasts and in human melanocytes.

### In *tfap2a* mutant embryos, *kita* expression is reduced in early melanophores but normal at later stages

**Figure 2:**
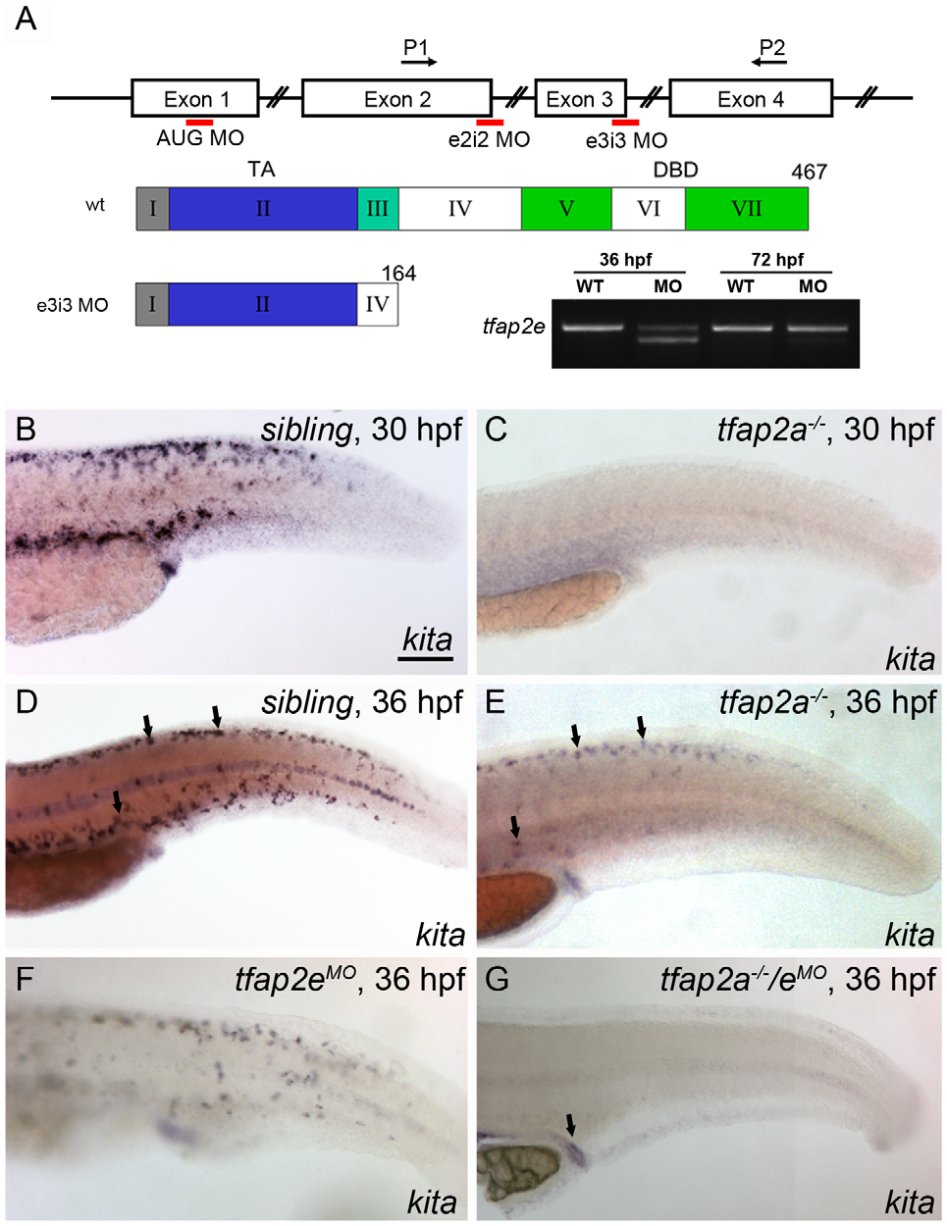
Expression of *kita* in melanophores is dependent on Tfap2a and Tfap2e. (A) Top, schematic of the *tfap2e* gene, showing the target sites of the MOs used in this study; middle, schematic indicating the effects of the *tfap2e e3i3* MO on the *tfap2e* transcript (Roman numerals refer to exons, other numbers refer to amino acids). TA, transactivation domain. DBD, DNA binding domain. The *e2i2* MO overlaps the exon 2 splice donor site, the *e3i3* MO overlaps the exon 3 splice donor site, and the *AUG* MO overlaps the translation start site, as indicated in red. P1 and P2 are the primers used for RT-PCR. *tfap2e e3i3* causes precise deletion of exon 3, leading to a frame shift and premature stop codon near the beginning of exon 4. Bottom right, Ethidium bromide-stained gel of PCR products generated from cDNA harvested from *tfap2e e3i3* MO injected embryos at the indicted stages. The MO has largely lost efficacy by 72 hpf. (B–G) Lateral views of embryos processed to reveal *kita* expression. (B,C) At 30 hpf, *kita* expression is readily detected in the dorsum of B) a sibling embryo but not C) a *tfap2a* mutant. (D, E) At 36 hpf *kita* expression is detectable in discrete cells in the dorsum of D) sibling embryos (arrows), E) *tfap2a* mutants (arrows), and F) sibling embryos injected with *tfap2e* MO, but is undetectable in G), the dorsum of *tfap2a* mutants injected with a *tfap2e* MO; *kita* expression is still detected in the cloaca of this last group (arrow). MOs are co-injected with *p53* MO in this and subsequent figures, to prevent cell death in the nervous system (see text). B) Scale bars: 100 µM (applies to B–G).

As discussed in the Introduction, *KITA* has been reported to be a direct target of TFAP2A, and a dominant negative AP2 variant was found to block *KIT* expression in cultured cells [18]; however the status of *kita* expression in *tfap2a* mutants has not been fully investigated. In zebrafish *tfap2a* mutants or *tfap2a* MO-injected embryos at 28 hpf, *kita* expression in the melanophore lineage is reduced to undetectable levels as assessed by *in situ* hybridization [24], [25]. However because melanophores undergo cell death in *kita* mutants but do not do so in *tfap2a* mutants, it has been proposed that *kita* is expressed in the melanophore lineage of *tfap2a* mutants at a later stage [24]. To test this prediction, we crossed heterozygote *tfap2a* null mutants (i.e., *lockjaw*, *tfap2a^ts213^*) and identified homozygous mutant offspring (hereafter, *tfap2a* mutants) at 28 hpf by virtue of their pigmentation phenotype. We fixed a fraction of these embryos at 28 hpf, and incubated the remainder in water containing phenylthiourea (PTU) to prevent melanin synthesis, until 36 hpf. We then processed all embryos to reveal *kita* expression. In *tfap2a* mutants at 28 hpf, *kita* expression in melanophores was undetectable by *in situ* hybridization (Figure 2C), as previously reported. However, at 36 hpf, *kita* expression was clearly visible in cells present in the dorsum of these embryos (Figure 2E). Thus normal *kita* expression in melanoblasts at 28 hpf is dependent on *tfap2a*, but later becomes independent of it. To explain these observations we hypothesized that Tfap2e compensates for the loss of Tfap2a and activates *kita* expression by 36 hpf.

To test whether Tfap2e maintains *kita* expression in *tfap2a* mutants, we first assessed *tfap2e* expression in *tfap2a* mutants, and found that it was expressed on schedule in migrating neural crest, as in wild-type embryos (Figure S2). Next we injected embryos with a morpholino (MO) targeting the *tfap2e* exon 3 splice donor site (i.e., *tfap2e e3i3* MO) (Figure 2A). To confirm the efficacy of this MO towards its intended target, we harvested RNA from embryos injected with the *tfap2e e3i3* MO, generated first-strand cDNA, and performed PCR using primers in exon 1 and exon 4. Sequencing of the major aberrant splice product revealed that the *e3i3* MO causes deletion of exon 3 in its entirety, resulting in a frame shift and a severe truncation of the predicted protein that eliminates the DNA binding domain (Figure 2A). By semi-quantitative PCR, this MO appears to inhibit normal splicing of the majority of *tfap2e* transcripts at 36 hpf, but to act with greatly reduced efficiency at 3 days post fertilization (dpf) (Figure 2A). By 24 hpf, wild-type zebrafish embryos injected with *tfap2e e3i3* MO showed evidence of cell death in the central nervous system (CNS), i.e., patches of opacity in the brain and spinal cord, but no other gross morphological defects; possibly this was due to non-specific toxicity of the MO to the embryo. Despite this cell death, the melanophores that developed in such embryos looked normal and were normally distributed (see below and Figure S3). *tfap2e e3i3* MO-induced CNS cell death was reduced by co-injection of *p53* MO, implying that Tfap2e has a role in cell survival in the CNS, or that the *tfap2e e3i3* MO has non-specific toxicity towards the nervous system, which is true of many MOs (Figure S3) [35]. To preserve the morphology of embryos, in all experiments discussed hereafter we have included *p53* MO with *tfap2e e3i3* MO. Interestingly, in *tfap2a* mutants injected with the *tfap2e e3i3* MO (hereafter, *tfap2a/e* doubly-deficient embryos), *kita* was absent from the dorsum at 36 hpf, although *kita* expression was readily detected in the cloaca and pharyngeal pouches (Figure 2G and not shown). These findings imply that in absence of Tfap2a, Tfap2e promotes *kita* expression in the melanophore lineage.

### Simultaneous reduction of Tfap2a and Tfap2e inhibits melanophore development

**Figure 3:**
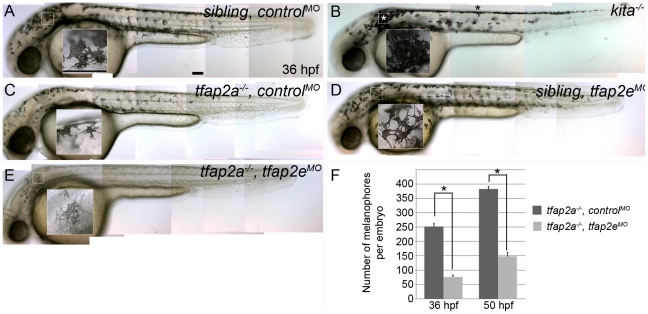
*tfap2e* morpholino has no effect in wild-type embryos, but disrupts melanophore differentiation in *tfap2a* mutants. (A–E) Lateral views of live embryos at 36 hpf. (A) A sibling embryo injected with a negative control MO (*control^MO^*), with normal melanophores. (B) A *kita^b5^* homozygous mutant, in which melanophores remain in the trunk dorsum (black asterisk) and near the otic vesicle (white asterisk), but are normally melanized. (C) A *tfap2a^ts213^* homozygous mutant injected with a control MO (*tfap2a* **^−/−^**,*control* ^MO^), exhibiting fewer melanophores than siblings and wild type embryos. (D) A sibling embryo injected with *tfap2e e3i3 MO* (*tfap2e* ^MO^), with melanophores that are normal with respect to both number and differentiation. The pictured melanophore appears to be more spindly than control counterparts, but this was not a reproducible effect. (E) A *tfap2e MO-*injected, presumed *tfap2a* mutant embryo (*tfap2a* **^−/−^**/*tfap2e^MO^*), showing fewer melanin-producing melanophores than in *tfap2a* mutants (82 of 312 injected embryos from an incross of heterozygous *tfap2a* mutant fish resembled the pictured embryo). (F) Histogram presenting the average number (± standard error, SE) of pigmented melanophores per *tfap2a* **^−/−^/** *control^MO^* and *tfap2a* **^−/−^**/tfap2*e* ^MO^ embryo at 36 hpf and 50 hpf. n = 10 embryos, asterisks indicate a p value <0.05. Scale bars: 100 µm.

Because of the sustained loss of *kita* expression in *tfap2a/e* doubly-deficient embryos, we expected that the phenotype in these embryos would be similar to that of *kita* homozygous null mutants, although perhaps not as severe because MO-mediated inhibition of gene expression is transient and partial; instead, however, we detected a much more severe phenotype. At 36 hpf, compared to the embryonic melanophores in their non-mutant siblings (Figure 3A), those in *kita* null mutants (i.e., *kita^b5^*) (Figure 3B) appeared normally melanized, but were reduced to about 60% of their normal numbers (because of a presumed defect in cell division) and did not migrate as extensively as their wild-type counterparts [23], [36]. In control MO-injected *tfap2a* mutants (Figure 3C), embryonic melanophores exhibited these same phenotypes. In *tfap2e* MO-injected sibling embryos (Figure 3D) there was no apparent melanophore phenotype. However, in *tfap2a/e* doubly-deficient embryos there were far fewer melanophores than present in control MO-injected *tfap2a* mutant embryos. Compared with control MO-injected *tfap2a* mutants, *tfap2a/e* doubly-deficient embryos had fewer pigmented melanophores in the dorsum and almost no visible melanophores on the lateral sides of the trunk or on the yolk sac (Figure 3E); this difference was still apparent at 84 hpf (not shown). In summary, whereas wild-type embryos injected with the *tfap2e* MO developed normally until at least 4 dpf, *tfap2a/e* doubly-deficient embryos displayed melanophore defects more severe than those of *tfap2a* or *kita* mutants. These findings suggest that Tfap2a and Tfap2e have partially redundant function in zebrafish melanophore development, and that this function exceeds the simple maintenance of *kita* expression.

To confirm the specificity of the *tfap2e e3i3* MO-induced melanophore phenotypes, we co-injected mRNA encoding a glucocorticoid-fused version of Tfpa2a *(tfap2aGR*), whose nuclear transport is dexamethasone-inducible, or *lacZ* as a control, into embryos injected with MOs targeting *tfap2a, tfap2e*, and *p53* (hereafter also termed *tfap2a/e* doubly-deficient embryos). Dexamethasone was added to both groups at 70% epiboly to avoid gastrulation defects caused by *tfap2a* over-expression [28]. Embryos were then scored for the rescue of under-melanized melanophores, seen in *tfap2a/e* doubly-deficient embryos, at 36 hpf. We found that *tfap2aGR* mRNA effectively rescued melanophores in *tfap2a/e* doubly-deficient embryos, whereas *lacZ* did not (Figure S4G and S4H). As an alternative approach for testing specificity, we purchased two additional independent *tfap2e* MOs—one targeting the exon 2 splice donor site (i.e., *e2i2* MO) and the other the translation start site of the *tfap2e* gene (i.e., *AUG* MO) (Figure 2A). Injection of either the *tfap2e e2i2* MO or the *tfap2e AUG* MO into wild-type embryos had no effect on melanophore development, although both induced some degree of nervous-system cell death. Upon injection of either the *tfap2e e2i2* MO or *tfap2e AUG* MO into embryos derived from *tfap2a* mutant heterozygous parents, about one fourth of embryos exhibited the melanophore phenotype seen with the *tfap2e e3i3* MO (Figure S4A-S4F); co-injection of *p53* MO did not alter the melanophore phenotypes although it reduced nervous system cell death (not shown). These multiple tests of specificity strongly argue that the melanophore phenotypes we observe in *tfap2e* MO-injected embryos result from inhibition of *tfap2e* expression and not from off target effects.

### Inhibition of *tfap2e* does not further reduce melanophore specification in *tfap2a* mutants

**Figure 4:**
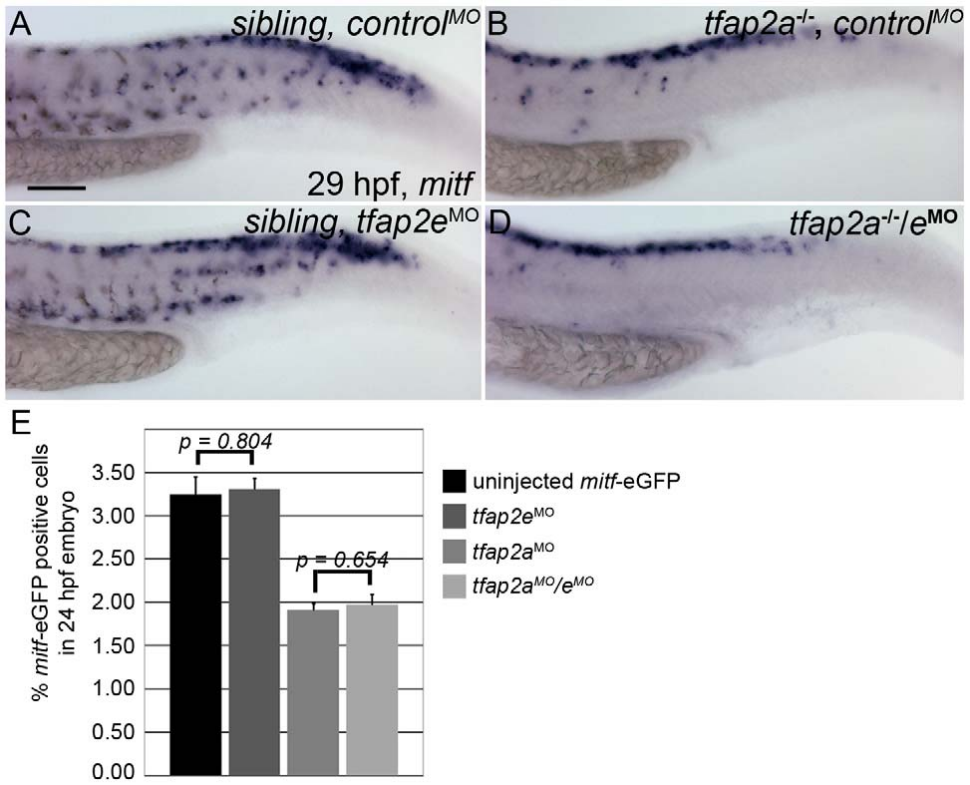
Numbers of *mitfa*-expressing cells are equivalent in *tfap2a/e* doubly-deficient and *tfap2a* deficient embryos. (A–D) Lateral trunk views of 29 hpf embryos of the indicated genotypes, injected with either control MO or *tfap2e e3i3* MO, as indicated, and processed to reveal *mitfa* expression. Relative to the sibling embryo shown in (A), the *tfap2a* **^−/−^** mutant embryo injected with a control MO (B) clearly has fewer cells expressing *mitfa*. (C) A *tfap2e^MO^* injected sibling embryo, with normal number of *mitfa* expressing cell numbers. (D) A *tfap2a* **^−/−^**/*e* ^MO^ embryo. The number of *mitfa* expressing cells is similar to that seen in *tfap2a^−/−^* mutants (N = 10 embryos, *tfap2a^−/−^* 287.2±5.8, *tfap2a^−/−^/^eMO^* 275.4±6.1, p = 0.18). The loss of *mitf*-expressing cells in *tfap2a* **^−/−^** and *tfap2a* **^−/−^**/*e* ^MO^ embryos is particularly prominent in the ventral portion of the tail. (E) Counts of GFP-expressing cells, scored by FACS, in dissociated 24 hpf *mitfa:GFP* transgenic embryos that were uninjected, injected with *tfap2a* MO, *tfap2e* MO, or *tfap2a/e* MO, as indicated. Numbers indicate the average (±SE) percentage of the GFP-expressing cells at 24 hpf; p values from a Student t-test are indicated. Bars one and two compare the percentage of *mitfa-*EGFP-positive cells from 24 hpf uninjected *mitfa-*EGFP transgenic embryos (*n*>50 embryos, 3 independent repeats) and 24 hpf *tfap2e* ^MO^ embryos (*n*>50 embryos, 3 independent repeats). Bars three and four compare the percentage of *mitfa-*EGFP-positive cells from 24 hpf *tfap2a* ^MO^ embryos (*n*>50 embryos, 3 independent repeats) and 24 hpf *tfap2a* ^MO^/*e* ^MO^ embryos (*n*>50 embryos, 3 independent repeats). Student t-test analysis indicates that there is no significant difference between the numbers of *mitfa-*EGFP-positive cells in *tfap2a*-deficient embryos and *tfap2a/e*-deficient embryos (*p* = 0.65). (A) Scale bar, 100 µm, applies to all panels.

The reduced number of melanophores in *tfap2a/e* doubly-deficient embryos relative to *tfap2a* mutants could reflect a role for Tfap2a/e activity in the specification of melanoblasts or, alternatively, in either survival or differentiation of melanophores. To distinguish among these possibilities, we examined the expression of *mitfa*, an early marker of the melanoblast and xanthoblast lineages [31], [32]. At 29 hpf, *mitfa*-expressing cells are visible in the head and trunk of wild-type embryos injected with a control MO (Figure 4A). The number of *mitfa*-expressing cells is reduced by about half in *tfap2a* mutant embryos injected with a control MO (Figure 4B); this reduction results at least in part from the absence of *kita* in such mutants at this stage, because melanophores are reduced by this amount in *kita* mutants [23], as are *mitfa*-expressing cells (our unpublished observations). In *tfap2e* MO-injected, wild-type embryos, the number of *mitfa* cells is not grossly different from that in control MO-injected, wild-type embryos (Figure 4C). Interestingly, in *tfap2a/e* doubly-deficient embryos, the number of *mitfa*-expressing cells did not appear to be further decreased relative to that in control MO-injected *tfap2a* mutants (Figure 4D). To confirm these impressions, we counted *mitfa*-expressing cells over the hind yolk (see Materials and Methods) at 24 hpf, and compared the results for *tfap2a* mutants injected with control MO versus those injected with *tfap2e* MO; we found no significant difference (See Figure 4 legend for numbers). In addition, we used fluorescence-activated cell sorting (FACS) to count GFP-positive cells in dissociated *mitfa:egfp* transgenic embryos injected with MOs, and this analysis supported our findings from histology [37]. Thus, GFP-expressing cells were similarly reduced in *tfap2a* MO-injected and *tfap2a/e* doubly-deficient *mitfa:egfp* embryos (i.e., to about 40% of the number in controls), although the number of differentiated melanophores in *tfap2a/e* doubly-deficient embryos was clearly reduced relative to that in *tfap2a* MO injected embryos (Figure 4E, histogram). These findings imply that Tfap2 activity, provided by the redundant actions of Tfap2a and Tfap2e, is involved in a step of melanophore development that occurs subsequent to specification of the *mitfa*-positive lineage.

### Tfap2a/e activity is required for melanophore differentiation

**Figure 5:**
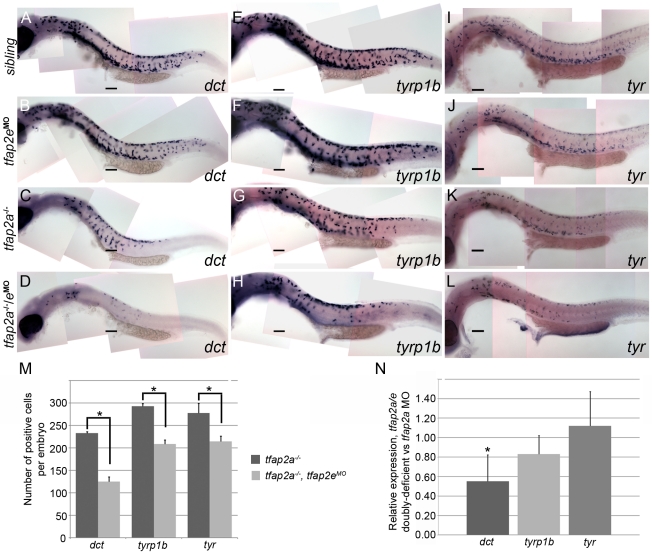
*tfap2a/e* doubl*y*-deficient embryos have defects in melanophore differentiation. (A–L) Lateral views of 29 hpf embryos processed to reveal (A–D) *dct*, (E–H) *tyrp1b*, and (I–L) *tyr* expression. (A, E, I) Sibling embryos have greater numbers of cells expressing these markers than do (C, G, K) *tfap2a* mutants; (D, H, L) in *tfap2a* **^−/−^**/*e* ^MO^ embryos a further reduction is apparent. This enhanced reduction is most apparent for *dct* expression. (B, F, J) Sibling embryos injected with the *tfap2e* MO resemble uninjected sibling embryos. Scale bars: 100 µm. (M) Histogram showing average number of *dct*-positive, *tyrp1b*-positive, and *tyr*-positive cells in the whole embryo at 29 hpf. First pair of bars, *dct*-positive cells in uninjected *tfap2a* **^−/−^** embryos (*n* = 10 embryos), vs. in *tfap2a* **^−/−^**/*e* ^MO^ embryos (*n* = 20 embryos); second pair of bars, *tyrp1b*-positive cells in the uninjected *tfap2a* **^−/−^** embryos (*n* = 10 embryos) vs. in *tfap2a* **^−/−^**/*e* ^MO^ embryos (*n* = 20 embryos). Final pair of bars, *tyr*-positive cells in uninjected *tfap2a* **^−/−^** embryos (*n* = 10 embryos) vs. in *tfap2a* **^−/−^**/*e* ^MO^ embryos (*n* = 10 embryos). Student t-test analyses indicate that the differences among cells expressing the indicated markers are statistically significant for *tfap2a*-deficient embryos vs. *tfap2a/e*-deficient embryos (for *dct*, *p* = 1.4×10^−9^; for *tyrp1b*, *p* = 1.5×10^−8^; for *tyr*, *p* = 0.02). (N) mRNA expression levels of differentiation markers in cells sorted from *mitf:egfp* embryos injected with the *tfap2a/e* MO (normalized to *β-actin*) relative to those in cells sorted from embryos injected with the *tfap2a* MO alone (normalized to *β-actin*) (* = p<0.05).

To determine which step in melanophore development depends on Tfap2 activity, we analyzed the expression of genes involved in melanophore differentiation: *tyr*, *tyrp1b* and *dct* [12]. In *tfap2a* mutant embryos at 29 hpf, the number of cells expressing each of these melanophore markers was reduced by about half relative to that in siblings, consistent with the previously described decrease in melanophores in *tfap2a* mutants (Figure 5A, 5E, 5I and 5C, 5G, 5K) [24], [25]. In *tfap2e* MO-injected embryos, the number of cells expressing each of these genes appeared to be normal (Figure 5B, 5F, and 5J), while in *tfap2a/e* doubly-deficient embryos their numbers were further reduced relative to that in *tfap2a* mutant embryos (Figure 5D, 5H, and 5L). To quantify this effect, we counted cells in embryos processed for *in situ* hybridization. We discovered that the reduction in gene expression was not equal in all cases. The number of cells expressing *dct* was most clearly and most consistently reduced in *tfap2a/e* doubly-deficient embryos, i.e., by approximately 47% relative to the number in *tfap2a* mutant embryos (Figure 5A-5D, and 5M). The reduction in *tyrp1b* and *tyr* expressing cells was more variable, with an average reduction of approximately 30% and 23%, respectively (Figure 5E-5L, and 5M).

The results described above indicate that when the expression of *tfap2a* and *tfap2e* is reduced, melanoblasts express *mitfa* but fail to progress to a stage at which they express normal levels of melanophore differentiation genes, such as *dct*, *tyrp1b*, and *tyr*. To test this model more quantitatively, we injected *mitfa:egfp* transgenic embryos [37] with either *tfap2a* MO or both *tfap2a MO* and *tfap2e* MO, dissociated them at 29 hpf, sorted and collected GFP-expressing cells, and measured the levels of various transcripts by quantitative RT-PCR (Figure 5N). Using this method, we saw a trend similar to that observed in the histology analysis: in GFP-positive cells sorted from *tfap2a/e* MO-injected embryos relative to those sorted from *tfap2a* MO-injected embryos, *dct* expression was reduced by approximately 45%, *tyrp1b* expression was reduced by 17%, and unexpectedly, *tyr* expression was not reduced. Taken together with the cell counts, these results reveal that Tfap2 activity, redundantly provided by Tfap2a and Tfap2e, promotes the differentiation of embryonic melanophores.

### Loss of Tfap2a/e activity does not result in a cell-fate switch or early cell death

**Figure 6:**
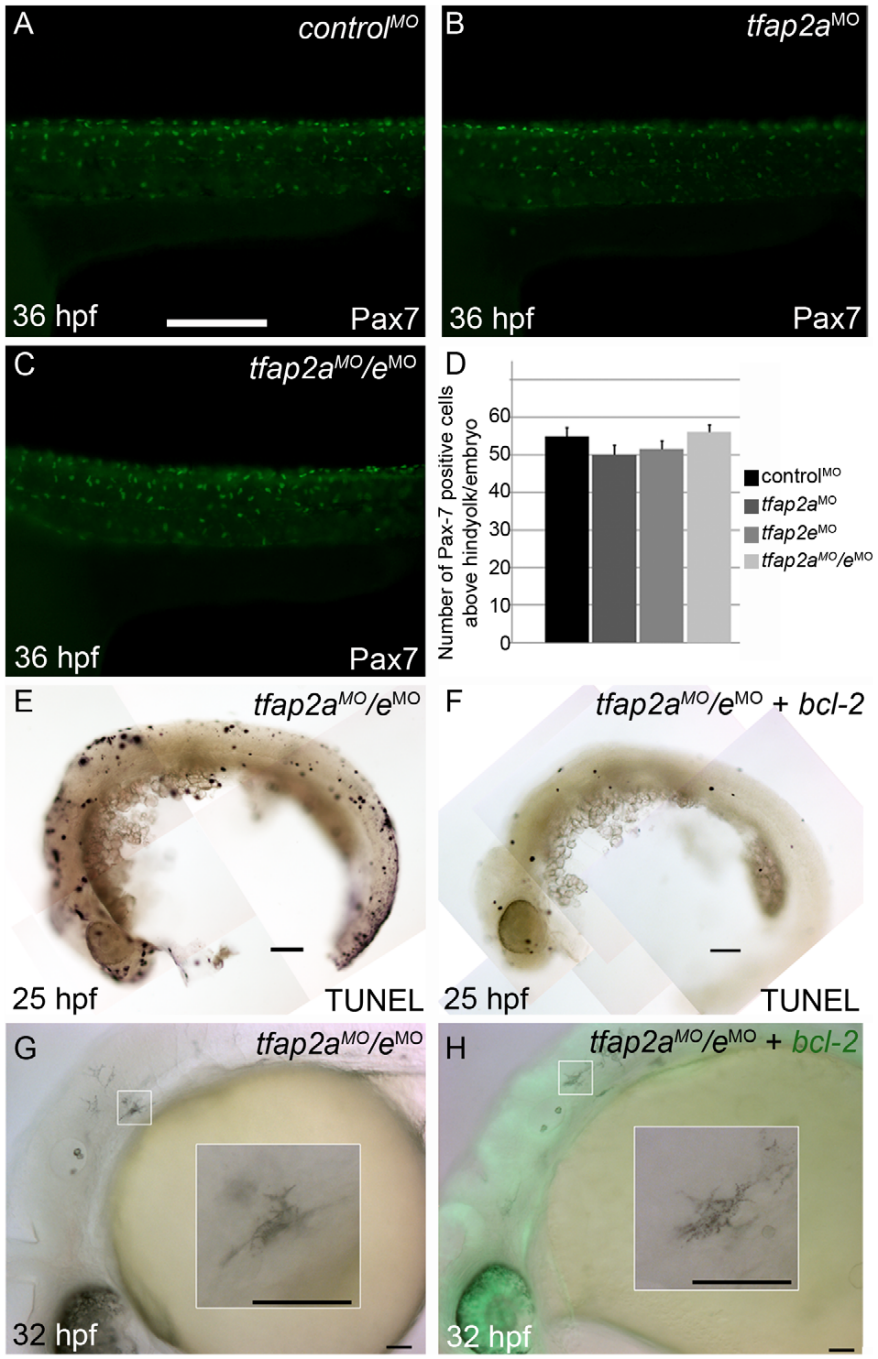
Contribution of cell fate specification and cell death to melanophore defects in *tfap2a/e* doubly-deficient embryos. (A–C) Lateral views of 36 hpf embryos stained with α-Pax7 to mark xanthophores. A similar number of α-Pax-7 IR positive cells is apparent in wild-type embryos injected with (A) a control MO, (B) the *tfap2a* MO, and (C) the *tfap2a* MO*/tfap2e* MO. (D) Average values for the number of α-Pax-7 IR positive cells counted above the hind yolk, n = 10 embryos per group. (E, F) Lateral views of 25 hpf embryos processed with the TUNEL reaction. (E) In an embryo injected with *tfap2a/e* MO alone there are many more TUNEL-positive cells than in (F) an embryo co-injected with an mRNA encoding a *bcl2-gfp* mRNA. (This effect was quantified in a parallel experiment, in which *bcl2GFP* mRNA was co-injected with control MO, embryos fixed at 24 hpf, and the number of TUNEL-positive cells counted: control MO, 97.7±15.5; control MO + *bcl2-gfp* 54.4±12.3, Avg±SE, p = 0.03). (G–H) Lateral views of live 32 hpf embryos. (G) In an embryo injected with the *tfap2a/e* MO alone, or (H) in an embryo co-injected with *bcl2-gfp* mRNA, melanophores appeared similarly poorly differentiated. Insets in G and H, higher magnification views of melanophores in the respective embryos. Scale bars: (A–C, E–H) 100 µM; (Insets in G–H) 50 µM.

We tested the possibility that the loss of differentiated melanophores in *tfap2a/e* doubly-deficient embryos results from a fate switch of melanophores to xanthophores, because *mitfa* is co-expressed with *c-fms*, a marker of xanthophore precursors [32]. We injected embryos with a control MO, *tfap2a* MO, *tfap2e* MO, or *tfap2a/e* MOs, and at 36 hpf processed them to reveal expression of anti Pax7 IR, a marker of xanthophores [33] (Figure 6A-6C and not shown). While the numbers of xanthophores in these groups did not differ significantly (Figure 6D), melanophore differentiation was clearly affected in *tfap2a/e* doubly-deficient embryos. These findings suggest that loss of Tfap2 activity in the melanophore lineage does not result in a cell fate switch.

We also assessed whether melanophores in *tfap2a/e* doubly-deficient embryos undergo cell death, i.e. despite the presence of *p53* MO. First, we co-injected embryos with MOs targeting *tfap2a* and *tfap2e* and with an mRNA encoding Bcl2, an inhibitor of apoptosis [38]. Injection of *bcl2* mRNA reduced the number of cells expressing a marker of programmed cell death in control embryos at 25 hpf (Figure 6E and 6F), but had no effect on the melanophore phenotype in *tfap2a/e* doubly-deficient embryos (Figure 6G and 6H). Secondly, embryos were incubated in acridine orange (AO), which is taken up by dying cells, from 16 hpf to 30 hpf and assessed for the presence of AO-containing cells in the dorsal neural tube and migratory neural crest. Relative to control MO-injected wild-type embryos, control MO-injected *tfap2a* mutants had an elevated number of such cells, but these numbers were not detectably increased in *tfap2e* MO-injected *tfap2a* mutants (data not shown). These findings suggest that loss of Tfap2 activity in melanophores does not result in either a switch in cell fate specification or promotion of cell death, but more likely in inhibition of normal melanophore differentiation.

### Tfap2a/e activity is cell-autonomously required for melanophore differentiation

**Figure 7:**
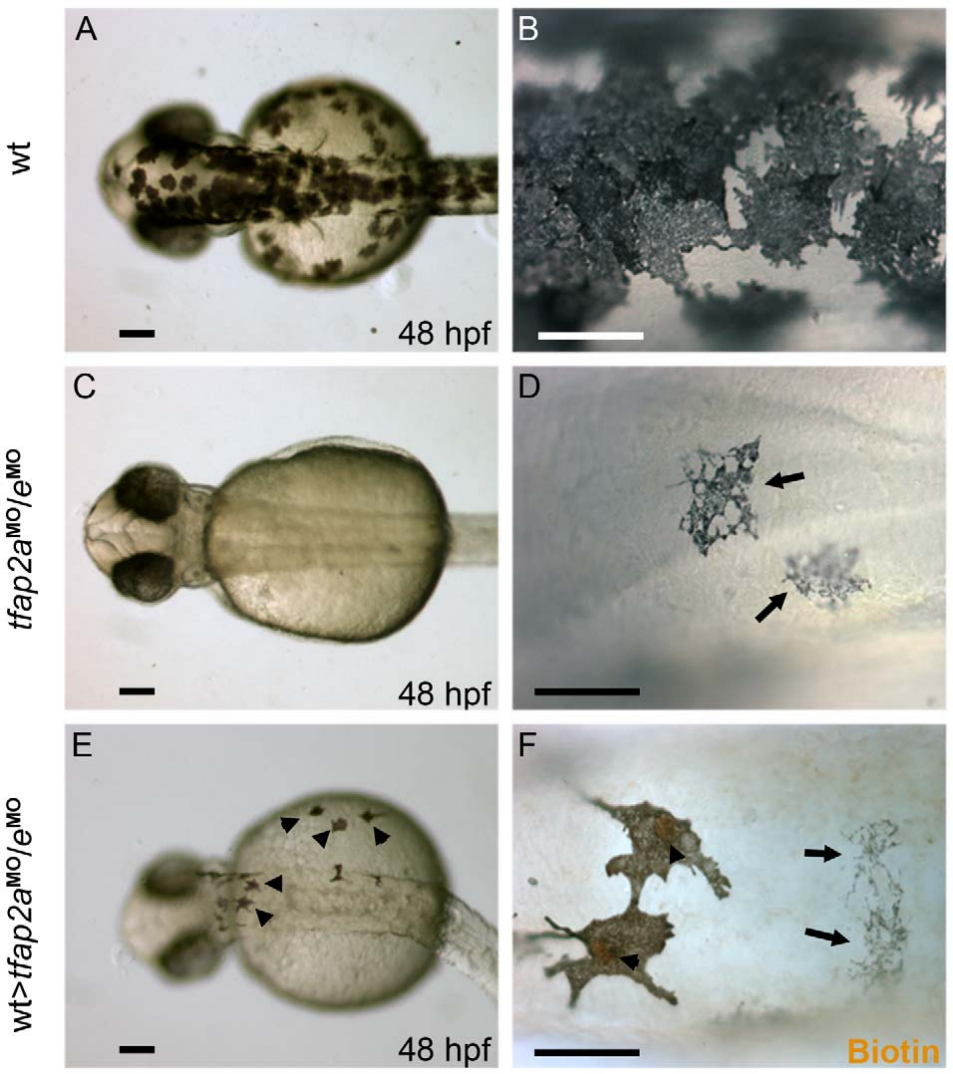
Tfap2a/e activity in melanophore differentiation appears to be cell-autonomous. (A–B) Dorsal views of a 48 hpf wild-type uninjected embryo, showing numerous, highly pigmented melanophores. (C–D) Dorsal views of a 48 hpf *tfap2a* ^MO^/*e* ^MO^ embryo. Numbers of melanophores, and the amount of melanin per melanophore, are reduced relative to control embryos. (E–F) Dorsal views of a 48 hpf chimera generated by transplanting cells from a wild-type donor injected with biotin dextran into a *tfap2a* ^MO^/*e* ^MO^ host, shown E) prior and F) subsequent to processing to reveal biotin. Arrowheads in E indicate normal looking melanophores. (F) Melanophores with two different morphologies are visible in this chimera. Normal-looking melanophores contain biotin (brown biotin label is most evident in the nuclei, arrowheads), indicating they are donor derived, while pale melanophores (arrows) lack biotin indicating they are host derived (In 4 embryos scored, 17 of 17 normal-looking melanophores were biotin-labeled). Scale bars: (A, C, E), 100 µm; (B, D, F), 50 µm.

In *tfap2a* mutants and MO-injected embryos, embryonic melanophores initially appear somewhat under-melanized [24], [25]. The *tfap2a* gene is expressed both in skin and neural crest, and we have reported evidence based on transplant studies that Tfap2a has both cell-autonomous and cell non-autonomous effects on melanophore differentiation [25]. Because *tfap2e* is expressed in melanoblasts but not skin, we assumed that the even poorer differentiation of melanophores in *tfap2a/e* doubly-deficient embryos is primarily a consequence of a cell autonomous role for Tfap2 activity. To confirm this prediction, we created genetic chimeras by carrying out transplantations at the blastula stage. Specifically, we transplanted cells from 4 hpf wild-type donors, which had been injected with a biotin-dextran as a lineage tracer, into 4 hpf hosts injected with *tfap2a/e* MO. We then reared the transplanted hosts to 48 hpf, and processed them for biotin staining to reveal the donor-derived cells. Melanophores lacking lineage tracer were indistinguishable from those seen in the untransplanted *tfap2a/e* MO-injected controls (Figure 7C-7F, arrows), whereas those positive for the lineage tracer were clearly darker, similar to wild-type controls (Figure 7A and 7B), indicating an increase in the level of melanin. In addition, they displayed a more normal morphology (Figure 7E and 7F, arrowheads). These findings indicate that normal melanophores can develop from wild-type cells that are flanked by *tfap2a/e*-deficient epidermis. This supports a cell-autonomous requirement for Tfap2a/e activity in melanophore differentiation.

### Forced *mitfa* expression partially restores melanophores in *tfap2a/e* doubly-deficient embryos

**Figure 8:**
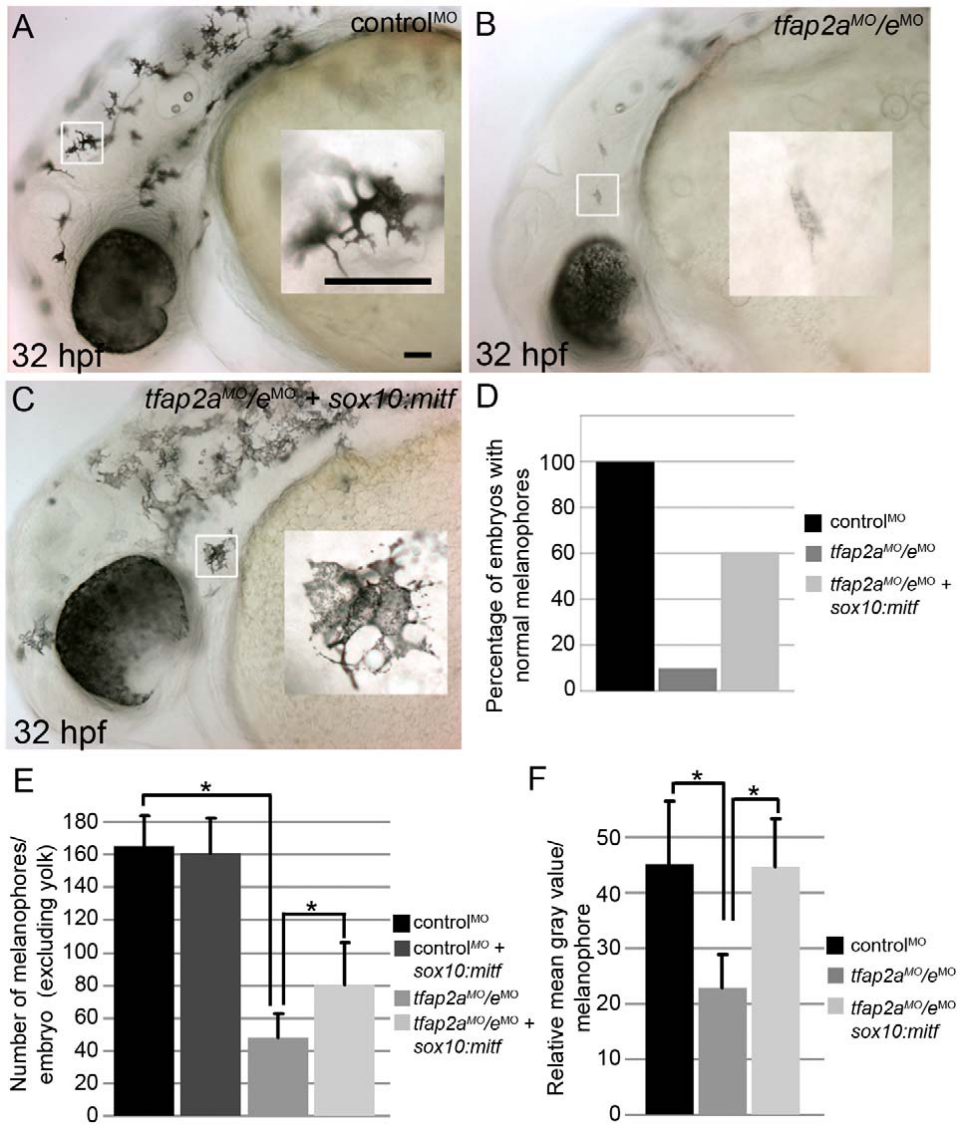
Melanophore differentiation in *tfap2a/e* doubly-deficient embryos is partially restored by forced expression of *mitfa*. (A–C) Lateral views of 32 hpf embryos, with anterior to the left. Insets are magnified images of the regions in white boxes. (A) A wild-type embryo injected with a control MO, exhibiting normal melanophores. (B) A wild-type embryo injected with *tfap2a* ^MO^/*e* ^MO^, exhibiting poorly melanized melanophores. (C) Wild-type embryo injected with *tfap2a* ^MO^/*e* ^MO^ and co-injected with *sox10*:*mitfa* plasmid; melanophores appear closer to normal in this embryo. (D) A histogram presenting percentage of embryos from the various groups with normal melanophores; n = 68 (control MO), 91 (*tfap2a/e* MO), 89 (*tfap2a/e* MO + *sox10:mitfa*), totaled from 3 independent experiments. Scale bars: 50 µM. (E) Histogram presenting average cell counts (±SE) of melanophores in embryos from various groups. Notice an increase in the number of melanophores in *tfap2a/e* MO embryos co-injected with *sox10:mitfa* compared to *tfap2a/e* MO alone (N = 10 embryos per group, asterisks indicate a p value <0.05). (F) Histogram representing average mean gray value (±SE), calculated with ImageJ analysis of photomicrographs of melanophores in indicated groups. Injection of *tfap2a/e* MO causes a reduction in the mean gray value of melanophores compared to that for control MO-injected wild-type embryos. This value is increased to wild-type levels upon co-injection of *sox10:mitfa* into *tfap2a/e* MO embryos (N = 10 embryos per group, approximately 70–80 melanophores per group, asterisks indicate p values <0.05).

Several signals are known to modulate Mitf transactivation activity [39], [40]. If Tfap2a/e is required for the expression of a component of such a signaling pathway, Mitfa activity might be reduced in *tfap2a/e* doubly-deficient embryos despite levels of *mitfa* mRNA being similar to those in *tfap2a* mutants. Alternatively, the Tfap2a/e effector required for melanocyte differentiation might be co-activated by Mitf. In either of these scenarios, forced *mitfa* expression might rescue melanophore differentiation in *tfap2a/e* doubly-deficient embryos. We injected *tfap2a/e* doubly-deficient embryos with a plasmid in which the *sox10* promoter drives *mitfa* expression (*sox10:mitfa)* [41], and found *sox10:mitfa*-injection increased the number of *tfap2a/e* doubly-deficient embryos with differentiated melanophores (compare Figure 8B to Figure 8C, 8D). We observed an increase in the number of darkly-pigmented melanophores in *tfap2a/e* doubly-deficient embryos injected with *sox10:mitfa* compared to in *tfap2a/e* doubly-deficient embryos alone (Figure 8E). We also quantified the mean gray value of single melanophores in these embryos (as a measure of pigment density), within a defined region, using ImageJ software. We found that there was a significant reduction in the pigment density of *tfap2a/e* doubly-deficient embryo melanophores, compared to control MO-injected embryo melanophores, and that this density was restored in doubly-deficient embryos co-injected with *sox10:mitfa* (Figure 8F).

Since *sox10* is expressed throughout the neural crest, we considered the possibility that *sox10:mitfa* might induce a conversion of neural crest to the melanoblast lineage, and that if this were to occur in neural crest that expressed another Tfap2 family member, normally differentiated melanophores might emerge in *tfap2a/e* doubly deficient embryos. However, arguing against this alternative model, we did not detect an increase in the number of melanophores in control-MO injected embryos co-injected with the *sox10:mitfa* plasmid (Figure 8E). Moreover, in this alternative model, *tfap2b* is the best candidate Tfap2 family member, as it is expressed in Rohon Beard sensory neurons [27], which are closely related to trunk neural crest [42], [43]. However, we found that even in embryos triply depleted of *tfap2a/b/e* using MOs, co-injection of *sox10:mitfa* plasmid elevated the number of normal-looking melanophores (our unpublished observation). Together these observations support the model that over-expression of *mitfa* can compensate for the role in melanophore differentiation normally played by Tfap2a/e, implying that the effector of Tfap2a/e-type activity necessary for melanophore differentiation acts upstream or in parallel with Mitfa.

## Discussion

### The phenotype of *tfap2a/e* double-knockdown embryos reflects multiple roles of Tfap2 activity in the melanophore lineage

Here we have presented two new findings relevant to the gene-regulatory-network (GRN) that governs the differentiation of zebrafish embryonic melanophores. First, *kita* expression in embryonic melanophores is positively regulated by Tfap2e, at least when Tfap2a levels have been reduced. Expression of *tfap2a* is present throughout the neural crest starting at the neurula stage, while the expression of *tfap2e* starts at approximately the time of neural crest delamination and appears to be restricted to melanoblasts [24], [25]. The relative timing of *tfap2a* and *tfap2e* expression explains why *kita* expression (in melanophores) in *tfap2a* mutants is reduced at 28 hpf, but present at later stages; Tfap2e compensates for the absence of Tfap2a but only after 28 hpf. The presence of *TFAP2E* expression in human melanocytes suggests that TFAP2A and TFAP2E have redundant or partially redundant function in mammalian melanocytes, as in fish melanophores. If so it would explain the observation, mentioned in the Introduction, that the coat color phenotype in mice with neural crest-specific deletion of *Tfap2a* is less severe than that of *Kit* homozygous null mutants [21].

The second unexpected finding is that Tfap2 activity (provided by Tfap2a and Tfap2e) promotes the differentiation of embryonic melanophores. This was revealed by reduced expression of the *dct* and *tyrp1b* mRNAs, as well as of melanin—changes that are evident in *tfap2a* mutants and more pronounced in *tfap2a/e* doubly-deficient embryos. Does Tfap2 activity also direct neural crest cells to join the melanophore sublineage? There is precedent for such a possibility, because Tfap2 activity provided by Tfap2a and Tfap2c appears to direct ectodermal precursors to join the neural crest lineage [28], [44]. In *tfap2a* single mutants, neural crest induction appears to occur normally, but *mitfa*-expressing cells, which are primarily melanoblasts, are reduced in number. This reduction may reflect a role for Tfap2 in melanophore specification or alternatively a reduction of Kita-mediated proliferation of melanoblasts. Whatever the explanation for reduced melanoblasts in *tfap2a* mutants, simultaneous reduction of *tfap2a* and *tfap2e* leads to a further reduction of melanophore numbers without a further reduction of *mitfa*-expressing cells, arguing Tfap2 promotes differentiation of melanoblasts to melanophores. While a reduction of melanophores without a reduction in *mitfa*-expressing cells might have been consistent with a cell fate change of melanophores to xanthophores (because markers of melanoblasts and xanthoblasts are briefly co-expressed [32]), xanthophore numbers are equivalent in *tfap2a* deficient and *tfap2a/e* doubly-deficient embryos, arguing against such a fate transformation. Does Tfap2 also promote survival of melanophores? We did not detect evidence of cell death of melanophores shortly after their differentiation in *tfap2a/e* doubly-deficient embryos. We predict that in embryos permanently deprived of both Tfap2a and Tfap2e melanophores would die as a consequence of the absence of Kita. However, because melanophores persist for several days in *kita* mutants, and this is longer than MOs are effective (see Figure 2A), it will be necessary to isolate a *tfap2e* mutant to test this prediction. Together these observations reveal that Tfap2 activity has multiple roles in melanophore development, including promoting melanophore differentiation.

Another result that will be important to revisit when a *tfap2e* mutant is available is the apparent heightened Tfap2-dependence of *dct* expression relative to *tyr* expression. Consistent with differential regulation of these related genes, in mice, *Dct* expression appears prior to *Tyr* expression, and this has also been suggested to be the case in zebrafish [45], [46]. However, because we knock-down *tfap2e* expression with an MO, the stronger effect on *dct* expression relative to on *tyr* expression may simply reflect loss of MO effectiveness over time. There may be a similar explanation for the inconsistent findings regarding *tyr* expression between the RNA *in situ* hybridization and the quantitative RT-PCR analyses. The cell dissociation protocol required for quantitative RT-PCR introduces a delay in the analysis of gene expression relative to that obtained using the RNA *in situ* hybridization protocol, giving further time for the MO to lose efficacy. Nevertheless, these results reveal that Tfap2 activity, redundantly provided by Tfap2a and Tfap2e, promotes the differentiation of embryonic melanophores.

### Tfap2 and Mitfa may co-activate melanophore differentiation genes

**Figure 9:**
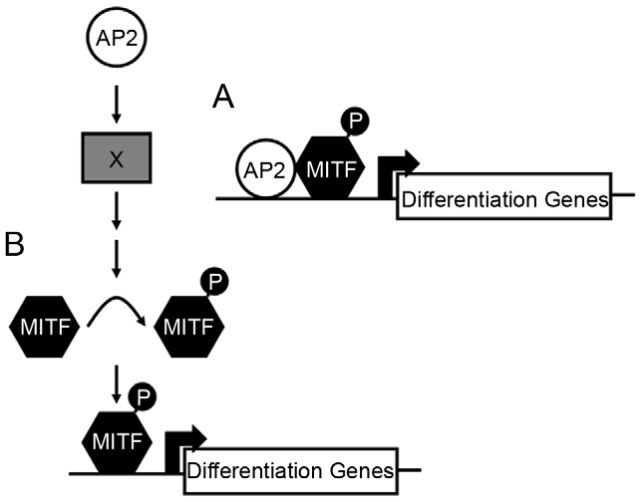
Potential models for how Tfap2 activity may function within the melanophore lineage. (A) In the first model, Tfap2 is a cofactor with Mitfa, and directly activates melanophore differentiation gene(s), such as *dct*. (B) In the second model, Tfap2 activity (provided by Tfap2a and Tfap2e) is upstream of an unknown factor (X), leading to modification of Mitfa, such as phosphorylation (denoted by “P”), and increased transactivation activity of Mitfa at target differentiation gene(s). In either scenario, forced expression of *mitfa* compensates for the loss of Tfap2 activity.

How does Tfap2 activity, mediated by Tfap2a and Tfap2e, effect melanophore differentiation? In *tfap2a/e* doubly-deficient embryos, melanophore differentiation fails but can be rescued by forced expression of *mitfa*. One model to explain these findings is that Mitfa and Tfap2 normally co-activate genes important for melanophore differentiation, but in the absence of Tfap2, elevated levels of Mitfa can suffice to do so (Figure 9A). Thus, Tfap2 family members may directly activate genes involved in melanin synthesis, such as *dct*, *tyrp1b*, and possibly *tyr,* all of which are known to be Mitfa targets [47]–[49]. Consistent with this possibility, recent studies have identified conserved DNA elements adjacent to the *dct* and *tyrp1b* genes that have melanocyte enhancer activity [13], and some of these contain putative Tfap2 binding sites. Simultaneous inhibition of *tyrp1a* and *tyrp1b* blocks melanization of zebrafish melanophores, suggesting that *tyrp1a/b* may partially mediate Tfap2a/e activity within these cells [50]. A variation of this model is that, rather than Tfap2 itself functioning as a co-activator with Mitfa, the protein product of a gene stimulated by Tfap2 does so. For instance, Tfap2 activates expression of estrogen receptor alpha (ERα) [51], [52]. ERα, together with p300, interacts with Mitf to strongly activate the *Dct* promoter [53].

### Tfap2 may indirectly promote Mitfa transactivation activity

It is also possible that the effector of Tfap2 activity is an enzyme that alters the activity, translation, or longevity of the Mitfa protein (Figure 9B). Thus, perhaps *mitfa* RNA levels are the same in *tfap2a* deficient vs. *tfap2a/e* deficient embryos, but Mitfa *activity* is reduced in the latter. For instance, the Tfap2-effector may be a receptor tyrosine kinase (RTK) whose activity results in posttranslational activation of Mitfa, i.e. similar to a proposed role of Kit [7], [8]. Supporting such a possibility, Kita itself is necessary for differentiation of embryonic melanophores in zebrafish in certain experimental conditions [54] [23]. A variety of RTKs are candidates for the Tfap2 effector in melanophore differentiation, including Erbb3 [55], [56], IGF1R [57], FGF receptor [58], c-Ret [59], and c-MET [60]. Two G-protein coupled receptors, which like RTKs can stimulate the MAP Kinase pathway, are also candidates. First, Endothelin receptor b (Ednrb signaling) promotes melanocyte differentiation in mammals, in part by activating MAP Kinase signaling and Mitfa [61]–[64]. While embryonic melanophores differentiate normally in zebrafish *ednrb1* mutants [65], uncharacterized *ednrb* homologues are present in the zebrafish genome (e.g., on chromosome 9) and may function in embryonic melanophores. Second, Melanocortin 1 receptor (Mc1r) is necessary for normal levels of pigmentation in zebrafish [66] and in mammals [67], and *MC1R* expression may be directly regulated by TFAP2A, because it has been shown that TFAP2A binds DNA adjacent to the *MC1R* gene in HeLa cells (chromatin immunoprecipitation results) [68]. Finally, Tfap2 could normally repress expression of an Mitfa phosphatase, alter processing of the *mitfa* transcript, change Mitfa translation or change Mitfa protein stability. All these scenarios would result in similar *mitfa* mRNA levels *in situ* but weaker Mitfa activity when Tfap2 levels are reduced, and would potentially be by-passed by over-expression of *mitfa* mRNA. The direct targets of Tfap2 in melanocytes are currently under investigation.

## Materials and Methods

### Fish maintenance

Zebrafish embryos and adults were reared as described previously [69], in the University of Iowa Zebrafish Facility. Embryos were staged by hours or days post fertilization at 28.5°C (hpf or dpf) [70]. Homozygous mutant embryos were generated from heterozygous adults harboring a presumed null allele of *tfap2a* (*lockjaw*, *tfap2a^ts213^*) [26], *mitfa* (*mitfa^b692^*) [31], or *kita* (*kita^b5^)* [23], as indicated.

### Generation of cDNAs and morpholinos

First-strand cDNA was synthesized from total RNA harvested from embryos at 4 hpf and 24 hpf as described [25]. A 1.4 kb full-length zebrafish *tfap2e* cDNA was amplified from the wild-type cDNA using the following primers: forward, 5′-GGA TTC ATG TTA GTC CAC TCC TAC TC-3′, reverse, 5′-TTA TTT GCG GTG CTT GAG CT-3′. This cDNA includes the entire open reading frame and was inserted into the pCR4-TOPO vector (Invitrogen, Carlsbad, CA). A 1.3 kb fragment of zebrafish *tyrp1b* cDNA was amplified from the wild-type 24 hpf cDNA using the following primers: forward, 5′-GAG AGC GGA TGA TAT AAG GAT GTG G-3′, reverse, 5′-GCC CAA TAG GAG CGT TTT CC-3′. This cDNA was inserted into pSC-A vector (Stratagene, La Jolla, CA).

In designing a *tfap2e* construct in which expression is disrupted, the exon 2 splice donor site and the exon 3 splice donor sites had to be inferred from comparison of the cDNA to the corresponding genomic sequence (http://uswest.ensembl.org/Danio_rerio/Info/Index). MOs complementary to these sites were ordered: *tfap2e e2i2* MO, 5′-ATA CAA GAG TGA TTG AAC TCA CCT G-3′; *tfap2e e3i3* MO, 5′-CAC ATG CAG ACT CTC ACC TTT CTT G-3′ (Gene Tools, Philomath, OR). In addition, a MO targeting the *tfap2e* translation start site (*AUG* MO) was designed, 5′-GCT GGA GTA GGA GTG GAC TAA CAT C-3′. MOs were reconstituted to 5 mg/ml in water and stored at room temperature (25°C). Immediately before use, they were diluted to 0.5 mg/ml in 0.2 M KCl. MOs (4–8 nl of diluted stock) were injected into the yolk underlying the blastomeres of embryos at the 1–4 cell stage. Upon injection of 3 ng or more of either MO, we saw evidence of non-specific toxicity, i.e., patches of opacity in the brain and spinal cord that did not develop when 5 ng of a *p53* MO (5′-GCG CCA TTG CTT TGC AAG AAT TG-3′) was injected [71]. To assure strong penetrance while preventing non-specific toxicity, we used 3 ng/embryo of *tfap2e e3i3* MO plus 5 ng/embryo of p53 MO to generate *tfap2a* ^−^/*e* ^MO^ embryos. For double MO experiments (*tfap2a^MO^/tfap2e^MO^)*, 3 ng of *tfap2e e3i3* MO, 5 ng *tfap2a e2i2* MO (5′-GAA ATT GCT TAC CTT TTT TGA TTA C-3′) and 5 ng of p53 MO were injected together. To test the efficacy of the *tfap2e* MOs, we used a pair of primers flanking a 305 bp fragment between exon 2 and exon 4 of *tfap2e* for RT-PCR (forward, 5′-CAC CAC GGC CTG GAT GAT ATT-3′; reverse, 5′-AGG ACT CCT CCA AGC AGC GA-3′). Additionally, where noted, a control MO (control^MO^) was used for comparison (5′-CCT CTT ACC TCA GTT ACA ATT TAT A-3′).

### Generating chimeric embryos

To create genetic chimeras, we injected donor embryos with 5 nl of 1% lysine-fixable biotinylated-dextran, 10,000 MW (Sigma, St. Louis, MO). At the sphere stage (4 hpf), about 100 cells were withdrawn from each donor embryo using a manual-drive syringe fitted with an oil-filled needle (Fine Science Tools, Vancouver, BC), and about 20 cells were inserted into each of several host embryos at the same stage. In placing these cells, we aimed for a position near the animal pole, to target clones to the ectoderm [72]. Host embryos were allowed to develop to 48 hpf, fixed, images taken, and then processed using an ABC kit (Vector Labs, Burlingame, CA) and DAB to reveal biotin as previously described [73], and subsequently photographed.

### Analysis of gene expression

The following restriction fragments were used to generate DIG-labeled antisense RNA probes (Roche Diagnostics, Mannheim, Germany) for whole mount *in situ* hybridization: *tfap2e*, NotI/T3; *tyrp1b*, BamHI/T3; *dct*, EcoRI/T7 [74]; *mitfa*, EcoRI/T7 [31]. Standard procedures were followed as previously described [75]. For total cell counts, 10–20 embryos were analyzed per group (see figure legend).

For immunohistochemistry, a monoclonal anti-Pax7 antibody [33] was used at a 1∶25 dilution (supernatant obtained from the Developmental Studies Hybridoma Bank at the University of Iowa, USA). The primary antibody and an anti-DIG antibody were added during routine whole mount *in situ* hybridization. Following development of whole mount *in situ* hybridization with NBT/BCIP, the embryos were blocked and then incubated with an Alexa-488 conjugated goat-anti-rabbit secondary antibody, as previously described [76]. After several washes, the embryos were mounted in 50% glycerol/PBST, and photographed. Cell counts were performed on ten embryos per group, along the entire length of the hind yolk.

### Dissociation of zebrafish embryos and FACS

Live embryos were reared to an appropriate stage, homogenized with a pestle, and dissociated with PBS containing trypsin and EDTA for 30 minutes at 33°C. After dissociation, cells were resuspended in PBS plus 3% fetal bovine serum (FBS). EGFP-positive cells were counted using a Becton Dickinson FACScan. For cell sorting, cells were dissociated as previously described, and subsequently sorted, on a Becton Dickinson FACS DiVa, directly into buffer RLT and β-mercaptoethanol for subsequent RNA isolation (RNeasy Plus Mini Kit, QIAGEN, Valencia, CA). FACScan cell counting, FACS DiVa cell sorting, and data analyses were conducted at the University of Iowa Flow Cytometry Facility.

### Quantitative RT-PCR

The isolation and culture of normal melanocytes and keratinocytes was performed as described previously, Mel 1 and Ker [77], [78], Mel 2,3 [79] (see Figure 1M). Total messenger RNA was isolated using an RNeasy Plus Mini Kit (QIAGEN, Valencia, CA), along with on-column DNase digestion according to the manufacturer's instructions. Lymphocytes (Jurkat cells, clone E6-1) were obtained (ATCC, Manassas, VA), and total RNA was isolated using the PerfectPure RNA Kit (following manufacturer's instructions, 5 PRIME Inc., Gaithersburg, MD). RNA concentrations were determined using a NanoDrop spectrophotometer (Thermo Scientific) and diluted to equal concentrations. For complementary DNA (cDNA) reactions, approximately 200 ng of total RNA was added to 0.5 µg random hexamers, plus 2.5 µl of 10 mM dNTPs (Invitrogen; Carlsbad, CA), and brought to 30 µl with nuclease-free water. Reactions were heated to 65°C for 5 minutes, and cooled to 4.0°C for 5 minutes in a PTC-200 Peltier Thermo Cycler (MJ Research; Ramsey, MN). We then added 19 µl of a master mix containing 10 µl of 5x First-Strand buffer (Invitrogen), 5 µl of 0.1 M dithiothreitol, 20 units of RNasin (Promega, Madison, WI), and nuclease-free water to a volume of 19 µl. Reactions were incubated at 25°C for 10 minutes, and then at 37°C for 2 minutes. Then 1 µl of Moloney-murine leukemia virus Reverse Transcriptase (New England Biolabs, Ipswich, MA) or 1 µl nuclease-free water was added to each reaction. Reactions were carried out at 37°C for 2 hours, followed by incubation at 75°C for 15 minutes. PCR reactions (25 µl) were prepared with approximately 10 ng of cDNA, using the SYBR Green kit (Applied Biosystems, Foster City, CA) following the manufacturer's instructions. The following primers were used at a final concentration of 200 nM in separate PCR reactions: human *TFAP2E* (forward: 5′-AAT GTG ACG CTG CTG ACT TC-3′; reverse: 5′-GGT CCT GAG CCA TCA AGT CT-3′); or human *GAPDH* (forward: 5′-AGG TCG GAG TCA ACG GAT TTG-3′; reverse: 5′-GTG ATG GCA TGG ACT GTG GT-3′). Quantitative real-time PCR in Low 96-well plates (Bio-Rad, Hercules, CA) was conducted using a Bio-Rad thermal cycler (CFX96 Real-Time PCR Detection System) and following the default protocol. Primers were designed to flank large exon-intron boundaries to avoid the potential amplification of contaminating genomic DNA. Also, RNA samples not reverse-transcribed (-RT) were used as a negative control. The 2^ΔΔCT^ method was used to determine relative levels of gene expression between samples (normalized to *GAPDH*) [80]. Experiments were performed in triplicate and mean and standard error were calculated. Following real-time PCR, melt-curve analysis was performed to determine reaction specificity. Similar methods were used for qRT-PCR of sorted cells, with the exception that approximately 20 ng of RNA was used for cDNA synthesis. The following primers were used at a final concentration of 200 nM in separate PCR reactions: *tyr* (forward: 5′-GGA TAC TTC ATG GTG CCC TT-3′; reverse: 5′-TCA GGA ACT CCT GCA CAA AC-3′); *tyrp1b* (forward: 5′-TAT GAG ACA CTG GGC ACC AT-3′; reverse: 5′-CAC CTG TGC CAT TGA GAA AC-3′); *dct* (forward: 5′-CCT CGA AGA ACT GGA CAA CA-3′; reverse: 5′- CAA CAC CAA CAC GAT CAA CA-3′); and *β-actin* (forward: 5′-CGC GCA GGA GAT GGG AAC C-3′; reverse: 5′-CAA CGG AAA CGC TCA TTG C-3′). Again, the 2^ΔΔCT^ method was used to determine relative levels of gene expression between samples, first normalizing both samples to *β-actin,* and then comparing relative gene expression levels in *tfap2a/e* doubly-deficient cells to those in *tfap2a* deficient cells.

### TUNEL staining

Apoptotic cell death was revealed in whole embryos by terminal transferase dUTP nick-end labeling (TUNEL) as described [81]. The terminal transferase reaction was terminated by incubation at 70°C for 30 min, and embryos were processed with anti-FITC-alkaline phosphatase antibody and developed with NBT/BCIP, as for an RNA *in situ* hybridization.

### Rescue experiments

For *tfap2aGR* mRNA rescue experiments, approximately 5 nL of 0.075 mg/mL *tfap2aGR* or *lacZ* encoding mRNA, transcribed *in vitro* (mMessage mMachine kit, Ambion, Austin, TX) was injected into one of four cells of embryos previously injected with *tfap2a/e/p53* MOs (similar concentration as indicated before). Embryos were raised until they reached approximately 75% epiboly, at which point dexamethasone (dissolved in EtOH) was added to the fish water at a final concentration of 40 µM. For DNA rescue experiments, 5 nL of a 0.025 mg/ml plasmid encoding 4.9 Kb of the *sox10* promoter driving full length *mitfa* [41] was injected at the one cell stage, followed by co-injection of various MO combinations (control MO and p53 MO or *tfap2a^MO^,tfape^MO^*). Embryos were then raised until approximately 36 hpf and fixed in 4% paraformaldehyde overnight. Finally, embryos were rinsed in PBST, mounted in 3% methylcellulose, and photographed.

### ImageJ analysis

To analyze the mean gray value of melanophores, embryos were first fixed at the appropriate stage in 4% paraformaldehyde overnight. Embryos were then rinsed in PBST and mounted in 3% methylcellulose, and images of single melanophores were taken near the otic vesicle at 40x. All lighting conditions remained constant throughout image capturing. 6–10 melanophores were imaged per embryo, and 10 embryos were analyzed per group (roughly 70–80 melanophores per group). Images were converted to a 32 bit gray image and then processed using the auto threshold function in ImageJ software (Version 1.40 g, National Institutes of Health, Bethesda, MD), creating an outline of the melanophore being analyzed. After application of the auto threshold function, a selection was created of the pixels highlighted, and a measurement reporting mean gray value for the given area was taken. An inverse of the selection was then created, highlighting the background (area not occupied by the melanophore), and a similar measurement was taken, reporting the mean gray value of the surrounding background. The difference was then calculated between the mean gray value of the melanophore and the surrounding background, resulting in the normalized mean gray value of the melanophore. Averages were then calculated for all melanophores measured per group, and standard deviation was calculated.

### Cell counts

For *mitfa*-positive and TUNEL-positive cell counts, the entire region overlying the hind yolk was counted. *For* melanophore cell counts in *sox10:mitfa* rescue experiments, the total number of melanophores in the embryo body (excluding yolk and hind yolk) were counted. Embryos were fixed in 4% paraformaldehyde overnight, washed in PBST, and mounted in 3% methylcellulose for counts. Embryos were mounted and then counted blindly by an independent observer.

## Supporting Information

Figure S1 Expression of *tfap2d* is absent from developing melanophores. Lateral views of wild-type zebrafish embryos, fixed at the stage indicated and processed to reveal *tfap2d* expression by RNA *in situ* hybridization. (A) At 24 hpf, an embryo shows *tfap2d* expression within specific regions of the midbrain, (B) which persists until 36 hpf. Importantly, *tfap2d* expression is not detected within the trunk of these embryos. Embryos were treated with low levels of PTU to decrease melanin production to allow better visualization of potential expression within melanophores. Scale bar: 50 µM.
(4.16 MB TIF)

Figure S2 Expression of *tfap2e* in *tfap2a* mutants. Lateral views of zebrafish embryos, fixed at the stage indicated and processed to reveal *tfap2e* expression by RNA *in situ* hybridization. (A) A sibling embryo at 28 hpf with *tfap2e* expression within melanoblasts, located throughout the trunk of the embryo, as described earlier. (B) A *tfap2a* mutant, in which *tfap2e* expression is detected within melanoblasts near the dorsum of the embryo; it is evident that fewer than normal numbers of *tfap2e*-expressing cells (presumed melanoblasts) have migrated ventrally. (C) Sibling and D) *tfap2a* mutant embryos at 34 hpf; *tfap2e* expression is detected in the posterior trunk of both sibling and mutant embryos, although fewer *tfap2e*-expressing cells have migrated ventrally in the *tfap2a* mutant. Embryos were treated with low levels of PTU to better visualize expression within melanophores. Scale bar: 25 µM.
(6.76 MB TIF)

Figure S3 *p53* MO blocks nervous system necrosis but does not affect melanophore development. Lateral views of live zebrafish embryos at 36 hpf. Insets show higher magnification of melanophores contained in white boxes. (A) A wild-type embryo shows normal melanophore development, similar to embryos injected with *tfap2e e3i3* MO (B,C). (B) The embryo injected with *tfap2e* MO also displays signs of central nervous system cell death (i.e., patches of opacity in the brain and spinal cord, white asterisk), which is reversed (C) by co-injection of a *p53* MO.
(7.25 MB TIF)

Figure S4 Specificity of *tfap2a/e* doubly-deficient melanophore defects. (A–F) Lateral views of live zebrafish embryos at 36 hpf. Insets show higher magnification of melanophores contained in the white boxes. (A–E) Sibling embryos injected with A), control MO, (C) *tfap2e e2i2* MO, or (E) *tfap2e AUG* MO; all of these embryos exhibit normally pigmented melanophores. (B) A *tfap2a* mutant embryo injected with a control MO, with a reduction in melanophore numbers and melanophore migration, and slightly less than normal melanization. (D,F) *tfap2a* mutant embryos injected with (D) a *tfap2e e2i2* MO or (F) a *tfap2e AUG* MO. These embryos display a further reduction in darkly pigmented melanophores, throughout the embryo. (G,H) Dorsal views of embryos at 36 hpf, anterior to the left. Embryos were first injected with *tfap2a/e MO*, followed by injection of mRNA encoding either (G) *lacZ* or (H) a dexamethasone-inducible version of tfap2a (*tfap2aGR*). Following injections, embryos were incubated in dexamethasone (Dex). The embryo injected with *tfap2aGR* shows rescue of pigmented melanophores whereas that injected with *lacZ* did not. Scale bars: 25 µM.
(7.40 MB TIF)
