# Supplementary figures and images for: Differentiation of Zebrafish Melanophores Depends on Transcription Factors AP2 Alpha and AP2 Epsilon
Source: PLoS Genet. 2010 Sep 16;6(9):e1001122. doi: 10.1371/journal.pgen.1001122 (PMC2940735; doi:10.1371/journal.pgen.1001122)

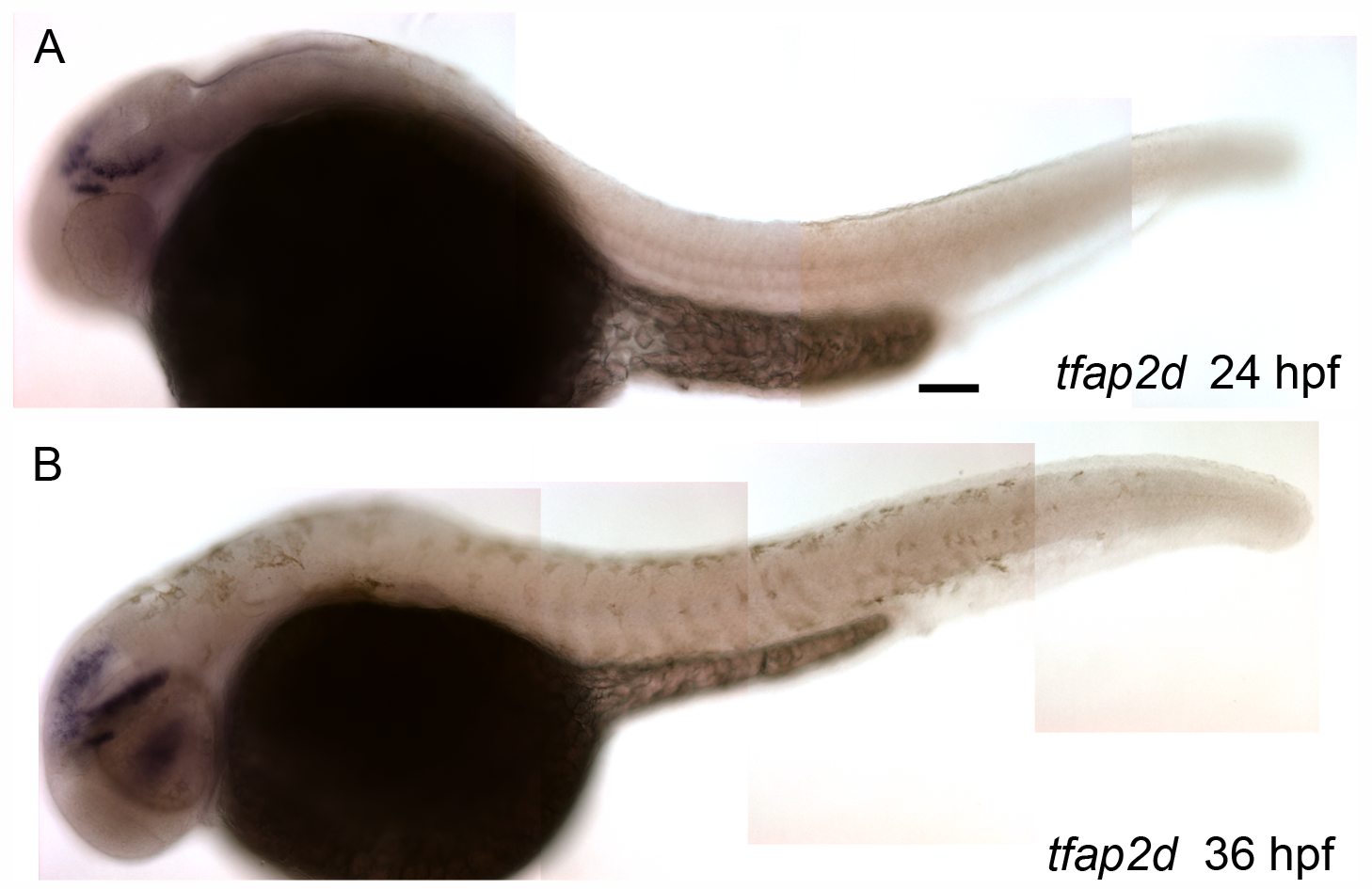

Supplement: Figure S1 — Expression of tfap2d is absent from developing melanophores. Lateral views of wild-type zebrafish embryos, fixed at the stage indicated and processed to reveal tfap2d expression by RNA in situ hybridization. (A) At 24 hpf, an embryo shows tfap2d expression within specific regions of the midbrain, (B) which persists until 36 hpf. Importantly, tfap2d expression is not detected within the trunk of these embryos. Embryos were treated with low levels of PTU to decrease melanin production to allow better visualization of potential expression within melanophores. Scale bar: 50 µM. (4.16 MB TIF) [file pgen.1001122.s001.tif]

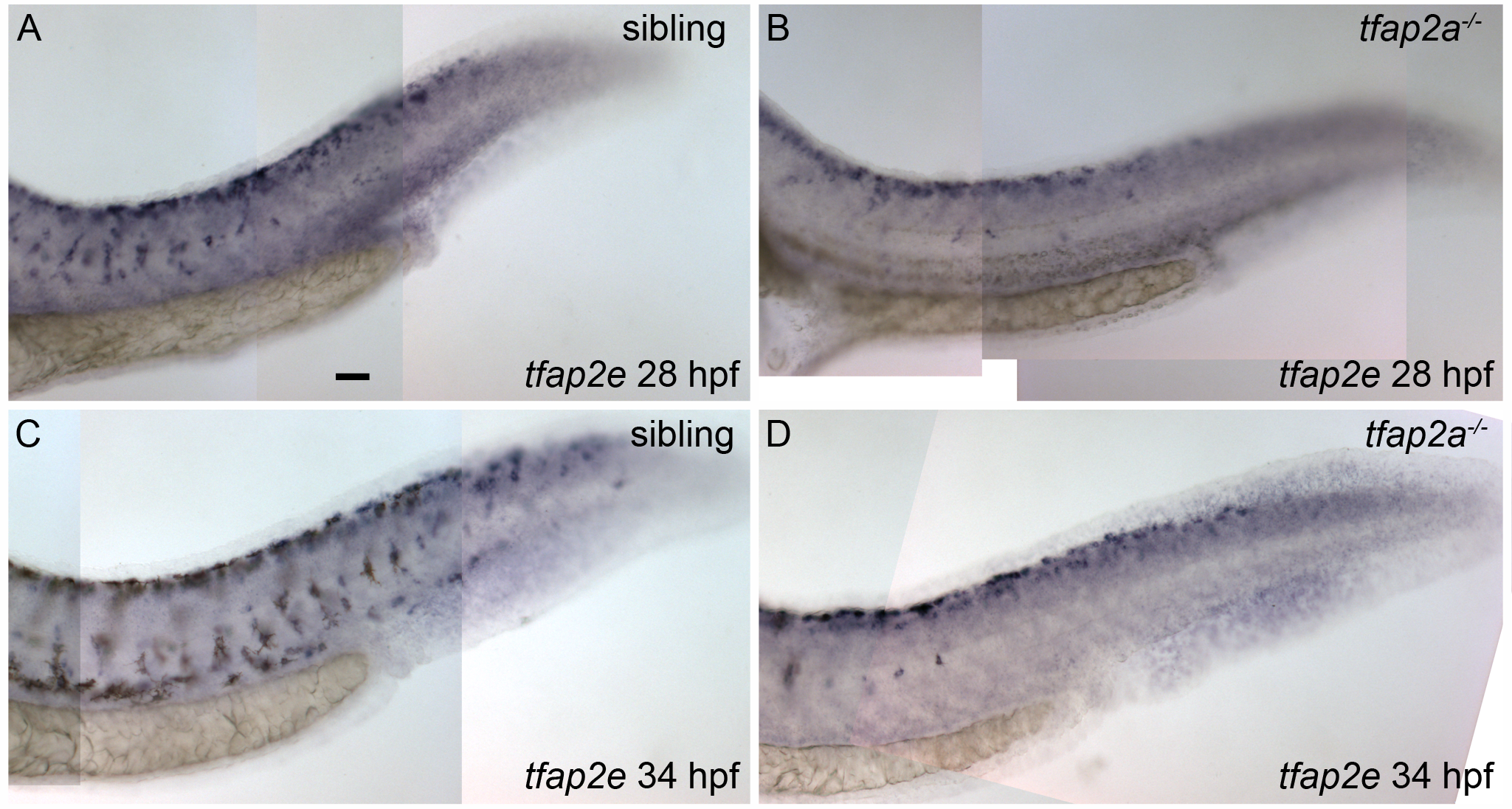

Supplement: Figure S2 — Expression of tfap2e in tfap2a mutants. Lateral views of zebrafish embryos, fixed at the stage indicated and processed to reveal tfap2e expression by RNA in situ hybridization. (A) A sibling embryo at 28 hpf with tfap2e expression within melanoblasts, located throughout the trunk of the embryo, as described earlier. (B) A tfap2a mutant, in which tfap2e expression is detected within melanoblasts near the dorsum of the embryo; it is evident that fewer than normal numbers of tfap2e-expressing cells (presumed melanoblasts) have migrated ventrally. (C) Sibling and D) tfap2a mutant embryos at 34 hpf; tfap2e expression is detected in the posterior trunk of both sibling and mutant embryos, although fewer tfap2e-expressing cells have migrated ventrally in the tfap2a mutant. Embryos were treated with low levels of PTU to better visualize expression within melanophores. Scale bar: 25 µM. (6.76 MB TIF) [file pgen.1001122.s002.tif]

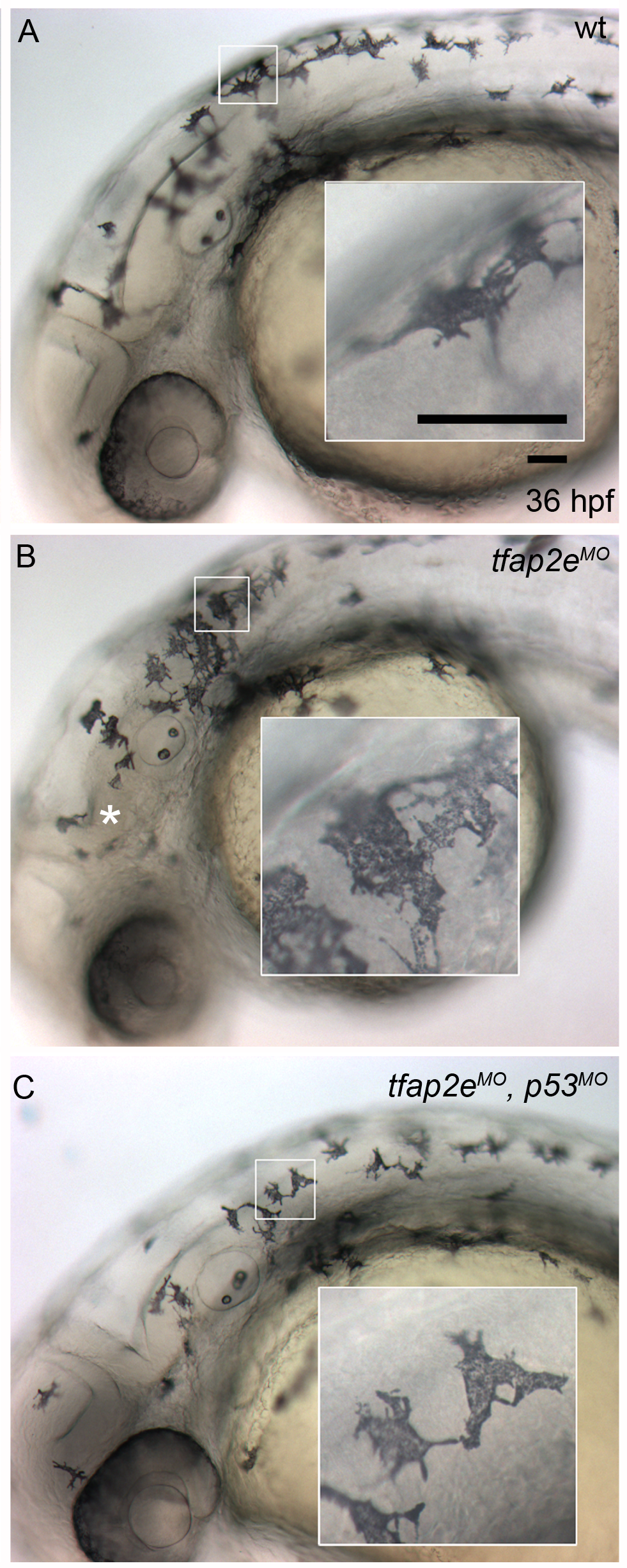

Supplement: Figure S3 — p53 MO blocks nervous system necrosis but does not affect melanophore development. Lateral views of live zebrafish embryos at 36 hpf. Insets show higher magnification of melanophores contained in white boxes. (A) A wild-type embryo shows normal melanophore development, similar to embryos injected with tfap2e e3i3 MO (B,C). (B) The embryo injected with tfap2e MO also displays signs of central nervous system cell death (i.e., patches of opacity in the brain and spinal cord, white asterisk), which is reversed (C) by co-injection of a p53 MO. (7.25 MB TIF) [file pgen.1001122.s003.tif]

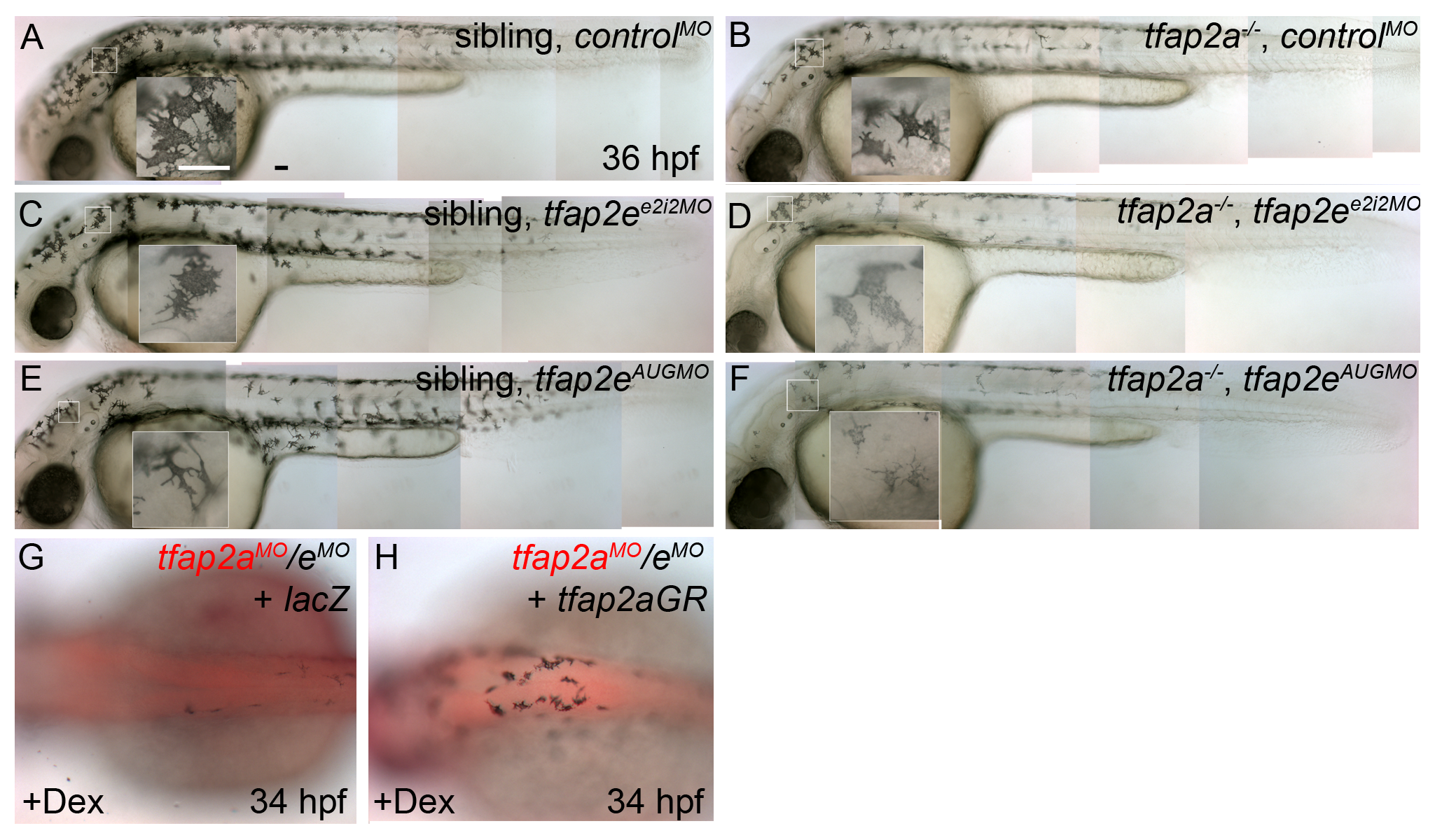

Supplement: Figure S4 — Specificity of tfap2a/e doubly-deficient melanophore defects. (A–F) Lateral views of live zebrafish embryos at 36 hpf. Insets show higher magnification of melanophores contained in the white boxes. (A–E) Sibling embryos injected with A), control MO, (C) tfap2e e2i2 MO, or (E) tfap2e AUG MO; all of these embryos exhibit normally pigmented melanophores. (B) A tfap2a mutant embryo injected with a control MO, with a reduction in melanophore numbers and melanophore migration, and slightly less than normal melanization. (D,F) tfap2a mutant embryos injected with (D) a tfap2e e2i2 MO or (F) a tfap2e AUG MO. These embryos display a further reduction in darkly pigmented melanophores, throughout the embryo. (G,H) Dorsal views of embryos at 36 hpf, anterior to the left. Embryos were first injected with tfap2a/e MO, followed by injection of mRNA encoding either (G) lacZ or (H) a dexamethasone-inducible version of tfap2a (tfap2aGR). Following injections, embryos were incubated in dexamethasone (Dex). The embryo injected with tfap2aGR shows rescue of pigmented melanophores whereas that injected with lacZ did not. Scale bars: 25 µM. (7.40 MB TIF) [file pgen.1001122.s004.tif]
